# Uncovering Beta-Lactam Susceptibility Patterns in Clinical Isolates of Mycobacterium tuberculosis through Whole-Genome Sequencing

**DOI:** 10.1128/spectrum.00674-22

**Published:** 2022-06-13

**Authors:** Francisco Olivença, Alexandra Nunes, Rita Macedo, David Pires, Cátia Silveiro, Elsa Anes, Maria Miragaia, João Paulo Gomes, Maria João Catalão

**Affiliations:** a Host-Pathogen Interactions, Research Institute for Medicines, Faculty of Pharmacy, Universidade de Lisboa, Lisbon, Portugal; b Bioinformatics Unit, Department of Infectious Diseases, National Institute of Health, Lisbon, Portugal; c National Reference Laboratory for Mycobacteria, Department of Infectious Diseases, National Institute of Health, Lisbon, Portugal; d Laboratory of Bacterial Evolution and Molecular Epidemiology, Instituto de Tecnologia Química e Biológica António Xavier, Universidade NOVA de Lisboa, Oeiras, Portugal; Johns Hopkins University School of Medicine

**Keywords:** *Mycobacterium tuberculosis*, drug resistance, antibiotic repurposing, beta-lactams, whole-genome sequencing

## Abstract

The increasing threat of drug resistance and a stagnated pipeline of novel therapeutics endanger the eradication of tuberculosis. Beta-lactams constitute promising additions to the current therapeutic arsenal and two carbapenems are included in group C of medicines recommended by the WHO for use in longer multidrug-resistant tuberculosis regimens. However, the determinants underlining diverse Mycobacterium tuberculosis phenotypes to beta-lactams remain largely undefined. To decipher these, we present a proof-of-concept study based on a large-scale beta-lactam susceptibility screening for 172 M. tuberculosis clinical isolates from Portugal, including 72 antimycobacterial drug-resistant strains. MICs were determined for multiple beta-lactams and strains were subjected to whole-genome sequencing to identify core-genome single-nucleotide variant-based profiles. Global and cell wall-targeted approaches were then followed to detect putative drivers of beta-lactam response. We found that drug-resistant strains were more susceptible to beta-lactams, but significant differences were not observed between distinct drug-resistance profiles. Sublineage 4.3.4.2 strains were significantly more susceptible to beta-lactams, while the contrary was observed for Beijing and 4.1.2.1 sublineages. While mutations in beta-lactamase or cell wall biosynthesis genes were uncommon, a rise in beta-lactam MICs was detected in parallel with the accumulation of mutations in peptidoglycan cross-linking or cell division genes. Finally, we exposed that putative beta-lactam resistance markers occurred in genes for which relevant roles in cell wall processes have been ascribed, such as *rpfC* or *pknA*. Genetic studies to validate the relevance of the identified mutations for beta-lactam susceptibility and further improvement of the phenotype-genotype associations are needed in the future.

**IMPORTANCE** Associations between differential M. tuberculosis beta-lactam phenotypes and preexisting antimycobacterial drug resistance, strain sublineage, or specific mutational patterns were established. Importantly, we reveal that highly drug-resistant isolates of sublineage 4.3.4.2 have an increased susceptibility to beta-lactams compared with other strains. Thus, directing beta-lactams to treat infections by specific M. tuberculosis strains and refraining its use from others emerges as a potentially important strategy to avoid resistance development. Individual mutations in *blaC* or genes encoding canonical beta-lactam targets, such as peptidoglycan transpeptidases, are infrequent and do not greatly impact the MICs of potent carbapenem plus clavulanic acid combinations. An improved understanding of the global effect of cumulative mutations in relevant gene sets for peptidoglycan and cell division processes on beta-lactam susceptibility is also provided.

## INTRODUCTION

Standard treatment for drug-susceptible tuberculosis (TB) requires the combined use of isoniazid, rifampicin, ethambutol, and pyrazinamide for several months. Incomplete or inconsistent treatment may favor the emergence of drug-resistant TB (DR-TB) strains. Multidrug-resistant TB (MDR-TB) is defined as a Mycobacterium tuberculosis strain resistant to, at least, isoniazid and rifampicin. A pre-extensively drug-resistant TB strain (pre-XDR-TB) is considered resistant to rifampicin and any fluoroquinolone, while extensively drug-resistant TB (XDR-TB) is attributed to a pre-XDR-TB isolate additionally resistant to, at least, bedaquiline or linezolid (https://www.who.int/publications/i/item/9789240037021). DR-TB is associated with poorer clinical outcomes and requires the use of less efficient and tolerable drugs. Hence, wider therapeutic options are desperately required to effectively treat DR-TB and beta-lactam antibiotics may offer a safe and prompt alternative (1, 2).

Beta-lactam exclusion from TB therapy is mainly attributed to an effective beta-lactamase, BlaC (3), and nonclassical peptidoglycan (PG) transpeptidases (4). Mycobacterial PG is mostly 3→3 cross-linked by L,d-transpeptidases, differing from the common 4→3 cross-links found in other bacteria, which are catalyzed by classical penicillin-binding proteins (PBPs). However, recent *in vitro* screenings suggest the susceptibility of clinical isolates of M. tuberculosis to beta-lactams (3, 5–10). A wide range of MICs is reported, suggesting a complex beta-lactam susceptibility spectrum, but knowledge of the phylogenetic or genetic determinants contributing to distinct M. tuberculosis beta-lactam phenotypes is scarce (7–9). In this context, we conducted a large-scale screening and whole-genome sequencing (WGS) to provide insight into the associations between antimycobacterial drug resistance, strain sublineage, or specific mutations with various levels of beta-lactam susceptibility.

## RESULTS

### Beta-lactam activity and clavulanate contribution.

**FIG 1:**
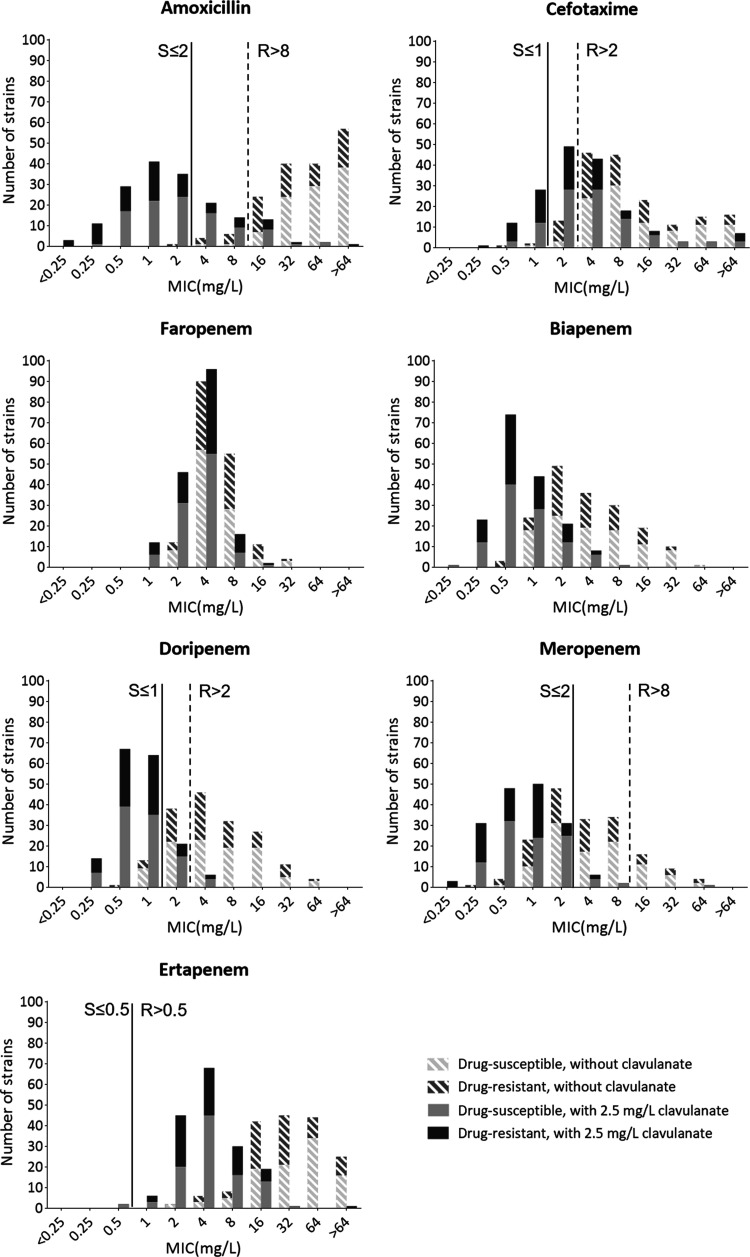
MICs of seven beta-lactams, with and without clavulanate, for 172 clinical M. tuberculosis strains. When present, clavulanate concentration was fixed at 2.5 mg/L. The vertical solid line delimits the MIC values below or equal to the susceptibility (S) breakpoint, while the dashed line marks the values above the resistance (R) breakpoint, based on EUCAST guidelines on PK-PD breakpoints (version 12.0), available for specific beta-lactams. CLSI or EUCAST do not provide these thresholds for biapenem or faropenem.

Beta-lactam susceptibility testing exposed a wide dispersion of MICs and indicated that the different antibiotics had distinct antimicrobial activity over M. tuberculosis strains (Fig. 1). Most of the isolates were resistant to amoxicillin (161/172) and cefotaxime (156/172), but 44% (76/172) were susceptible to meropenem (Table 1). Supplementation with clavulanate greatly potentiated beta-lactam efficacy and the percentages of beta-lactam/clavulanate susceptible strains were especially impressive among drug-resistant strains, reaching 76% (55/72) and 97% (70/72) for amoxicillin/clavulanate and meropenem/clavulanate, respectively. It was not possible to determine the proportion of susceptible strains to biapenem or faropenem because no clinical or pharmacokinetic-pharmacodynamic (PK-PD) breakpoints are defined in the Clinical and Laboratory Standards Institute (CLSI) or European Committee on Antimicrobial Susceptibility Testing (EUCAST) guidelines for these antibiotics.

**TABLE 1:** Geometric mean and susceptibility percentages of M. tuberculosis clinical isolates to beta-lactams, with and without clavulanate

Feature	AMX^*a*^	AMX/CLA	CTX	CTX/CLA	FAR	FAR/CLA	BIA	BIA/CLA	DOR	DOR/CLA	MEM	MEM/CLA	ETP	ETP/CLA
EUCAST nonspecies related breakpoints	S ≤ 2; R > 8	S ≤ 2; R > 8	S ≤ 1; R > 2	S ≤ 1; R > 2	-*^h^*	-	-	-	S ≤ 1; R > 2	S ≤ 1; R > 2	S ≤ 2; R > 8	S ≤ 2; R > 8	S ≤ 0.5; R > 0.5	S ≤ 0.5; R > 0.5
All clinical strains (172)^*b*^														
Beta-lactam susceptible	1 (0.6%)	119 (69.2%)	3 (1.7%)	41 (23.8%)	-	-	-	-	14 (8.1%)	145 (84.3%)	76 (44.2%)	163 (94.8%)	0 (0.0%)	2 (1.2%)
Beta-lactam intermediary	10 (5.8%)	35 (20.3%)	13 (7.6%)	49 (28.5%)	-	-	-	-	38 (22.1%)	21 (12.2%)	67 (39.0%)	8 (4.7%)	-	-
Beta-lactam resistant	161 (93.6%)	18 (10.5%)	156 (90.7%)	82 (47.7%)	-	-	-	-	120 (69.8%)	6 (3.5%)	29 (16.9%)	1 (0.6%)	172 (100.0%)	170 (98.8%)
Drug susceptible strains (100)^*b*^														
Beta-lactam susceptible	0 (0.0%)	64 (64.0%)	1 (1.0%)	15 (15.0%)	-	-	-	-	9 (9.0%)	81 (81.0%)	42 (42.0%)	93 (93.0%)	0 (0.0%)	2 (2.0%)
Beta-lactam intermediary	2 (2.0%)	25 (25.0%)	3 (3.0%)	28 (28.0%)	-	-	-	-	22 (22.0%)	15 (15.0%)	39 (39.0%)	6 (6.0%)	-	-
Beta-lactam resistant	98 (98.0%)	11 (11.0%)	96 (96.0%)	57 (57.0%)	-	-	-	-	69 (69.0%)	4 (4.0%)	19 (19.0%)	1 (1.0%)	100 (100.0%)	98 (98.0%)
Drug-resistant strains (72)^*b*^														
Beta-lactam susceptible	1 (1.4%)	55 (76.4%)	2 (2.8%)	26 (36.1%)	-	-	-	-	5 (6.9%)	64 (88.9%)	34 (47.2%)	70 (97.2%)	0 (0.0%)	0 (0.0%)
Beta-lactam intermediary	8 (11.1%)	10 (13.9%)	10 (13.9%)	21 (29.2%)	-	-	-	-	16 (22.2%)	6 (8.3%)	28 (38.9%)	2 (2.8%)	-	-
Beta-lactam resistant	63 (87.5%)	7 (9.7%)	60 (83.3%)	25 (34.7%)	-	-	-	-	51 (70.8%)	2 (2.8%)	10 (13.9%)	0 (0.0%)	72 (100.0%)	72 (100.0%)
Mean MIC^*c*^														
All clinical strains (172)	48.3	1.8	10.7	3.2	5.5	3.3	3.9	0.7	5.3	0.8	4.0	0.8	33.3	4.2
Drug-susceptible strains (100)	61.0	2.2	13.2	4.0	5.2	3.2	4.2	0.8	5.4	0.8	4.5	0.9	36.8	4.4
Drug-resistant strains (72)	34.9	1.3	8.0	2.3	5.9	3.4	3.6	0.7	5.0	0.7	3.4	0.6	29.1	4.0
Mono- or poly- resistant (14)	45.3	1.2	6.2	2.0	5.4	2.7	3.8	0.6	4.4	0.6	2.7	0.6	27.6	3.6
MDR (44)	31.0	1.3	8.5	2.4	6.0	3.6	3.5	0.7	5.2	0.8	3.6	0.6	29.4	4.1
Pre-XDR (14)	39.0	1.6	10.8	3.1	6.6	4.4	3.6	0.7	5.1	0.8	3.8	0.7	30.5	4.0
M. tuberculosis H37Rv WT^*d*^	64	2	8	2	4	2	4	0.5	4	0.5	2	0.5	32	4
Susceptible versus resistant^*e*^	4.16E−04	4.02E−03	7.38E−03	4.98E−04	7.70E−02	2.34E−01	5.35E−01	2.47E−01	6.72E−01	3.82E−01	1.09E−01	3.65E-03	3.20E−02	2.23E−01
Mono/poly versus MDR versus pre-XDR^*f*^	6.01E−01	6.35E−01	4.64E−01	6.30E−01	-	-	-	-	-	-	-	3.98E−01	1.00E+00	-
Beta-lactam:Beta-lactam/CLA Ratio*^g^*	27.5		3.3		1.7		5.5		6.8		5.2		7.9	

a AMX, amoxicillin; BIA, biapenem; CLA, clavulanate; CTX, cefotaxime; DOR, doripenem; ETP, ertapenem; FAR, faropenem; MEM, meropenem.

b Values for the global sample (172 strains) and specific drug-susceptible (100 strains) or drug-resistant (72 strains) subsets are displayed. Number and percentage of beta-lactam susceptible (S), intermediary, or resistant (R) strains, within each, considered set of strains, by definition from EUCAST guidelines on PK-PD breakpoints (version 12.0), available for specific beta-lactams.

c Geometric mean MIC values for each antibiotic condition, for the different strain subsets.

d Median beta-lactam MICs of three assays for M. tuberculosis H37Rv.

e Mann-Whitney U test *P* value obtained for the comparison between the MIC distributions of drug-susceptible and drug-resistant isolates. Values below 0.05 were considered significant.

f Kruskal-Wallis test *P* value obtained for the comparison between the MIC distributions of monoresistant or polyresistant, MDR, or pre-XDR isolates. Values below 0.05 were considered significant.

^g^Ratio represents the global mean MIC value without clavulanate divided by the MIC value with clavulanate.

^h^Dashes (-) signify not applicable.

The impact of clavulanate addition to the global geometric mean of a specific antibiotic was measured by calculating a beta-lactam:beta-lactam plus clavulanate ratio (Table 1). In accordance with the respective MIC distributions, this ratio was maximum for amoxicillin (27.5) and minimum for faropenem (1.67). The ratio was 3.3 for cefotaxime and ranged between 5.0 and 7.9 for carbapenems. Biapenem/clavulanate, doripenem/clavulanate, or meropenem/clavulanate were the most efficient combinations, all yielding mean MIC values between 0.7 and 0.8 mg/L. Despite not having the lowest values, faropenem MICs were very stable across the strain collection.

### Beta-lactam susceptibility and antimycobacterial drug resistance.

Drug-resistant strains consistently presented higher beta-lactam susceptibility percentages and lower mean MICs compared to their susceptible counterparts (Table 1). A Mann-Whitney U test confirmed statistically significant differences for amoxicillin, amoxicillin/clavulanate, cefotaxime, cefotaxime/clavulanate, meropenem/clavulanate and ertapenem (*P* < 0.05). To further investigate this relation, a Kruskal-Wallis test was performed between three resistance levels (monoresistant or polyresistant; MDR; pre-XDR), but no significant differences were found. In general, H37Rv is an adequate model for M. tuberculosis beta-lactam susceptibility because, for most antibiotics, the clinical global geometric mean was very close to the median MICs of the reference strain.

### Beta-lactam susceptibility and sublineage genotype.

**FIG 2:**
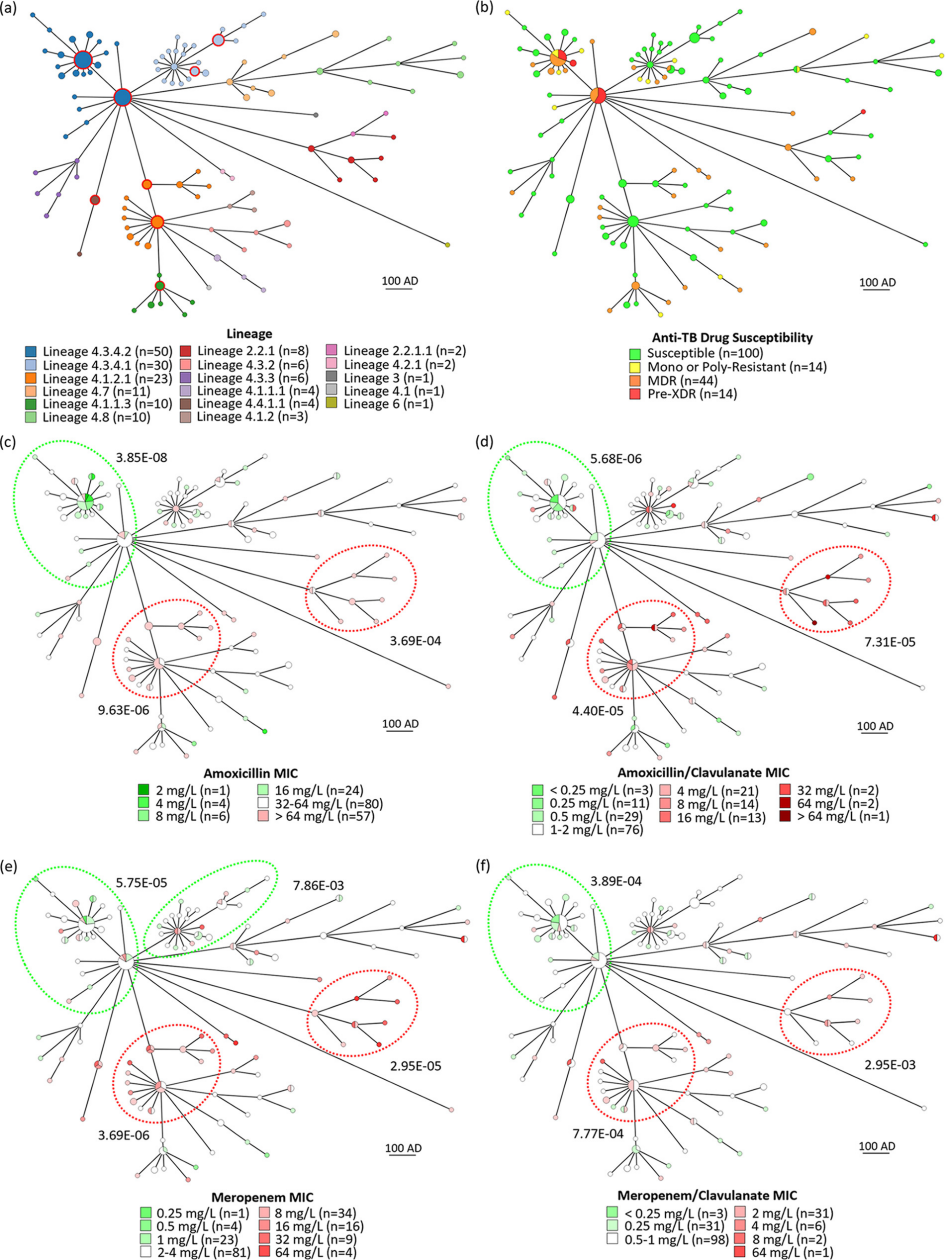
Minimum spanning trees generated for the 172 M. tuberculosis strains. The GrapeTree software (MSTree V2) was applied and strains sharing 12 or fewer variants collapsed in the same node. Node size and kurtosis are set to 100% while scaling is set to 300%. Branch length represents allelic differences (AD) between nodes. Nodes are colored according: (a) strain WGS lineage (nodes with more than two strains are labeled with a red circle); (b) anti-TB drug-resistance profile; (c) amoxicillin MIC; (d) amoxicillin/clavulanate MIC; (e) meropenem MIC; (f) meropenem/clavulanate MIC. Numbers between parentheses show the number of strains for a specific characteristic/condition. (c to f) Nodes are colored in shades of green or red according to the lower or higher MIC values of the correspondent strains. Strains with MICs immediately below and above the global geometric mean are in white. The dashed green or red circles define sublineages with significantly lower or higher MICs, respectively, compared with all other strains. Values next to the circles correspond to the respective comparison *P* value, obtained by the Mann-Whitney U test.

Among the clinical isolates that compose the sample (Fig. 2A; Table S1), 160/172 were members of lineage 4, which corresponds to Euro-American strains. Sublineages 4.3.4.2 (spoligotypes Latin American and Mediterranean 1 [LAM1], LAM4, and LAM 11) and 4.3.4.1 (spoligotypes LAM1 and LAM2) were the most common, with 50 and 30 strains, respectively. The 12 isolates that fell outside lineage 4 belonged to lineage 2 (East-Asian, all Beijing, *n* = 10), lineage 3 (East-African Indian, *n* = 1), and lineage 6 (Mycobacterium africanum West-African, *n* = 1). Regarding antimycobacterial drug resistance (Fig. 2B), 93% (13/14) of the pre-XDR strains were found within the 4.3.4.2 sublineage, while the other drug-resistance profiles were dispersed across sublineages. Differences in susceptibility to amoxicillin and meropenem, with and without clavulanate, were examined by the Mann-Whitney U test between each sublineage subset with more than 10 strains and all other strains (Fig. 2C to F; Table S2). Sublineage 4.3.4.2, comprised of 60% (30/50) of MDR/pre-XDR strains, presented significantly lower MICs to all treatments (*P* < 0.001). In sublineage 4.3.4.1 MICs were only significantly lower for meropenem (*P* < 0.01). On the other hand, significantly higher MICs were obtained for Beijing (*P* < 0.01) and 4.1.2.1 sublineage (*P* < 0.001) strains.

### Mutations in beta-lactamase or cell wall biosynthesis genes.

Core-genome single nucleotide variants (core-SNVs) in relevant genes for beta-lactam function or cell wall metabolism were examined to identify possible genomic determinants for beta-lactam susceptibility heterogeneity. After an extensive literature review, 53 chromosomal genes were selected and assorted into five categories: beta-lactamase activity (*n* = 3); PG synthesis (PG precursor production in the cytoplasm) (*n* = 11), PG assembly (PG cross-linking) (*n* = 12); PG hydrolysis (*n* = 13); cell division (*n* = 14). A total of 80 core-SNVs associated with nonsynonymous mutations were detected in 39/53 genes, with most of these mutations (56/80) being present in one or two strains (Table S3).

Focusing on mutations present in three or more isolates with a predicted functional deleterious effect (PROVEAN score <−2.5), it was possible to infer several associations with lower or higher MICs of amoxicillin and meropenem treatments (Table 2). Compared with the global mean, strains with the A49G substitution in BlaC (*n* = 4) had low mean MICs of amoxicillin and meropenem. The T188A substitution in PonA2 (*n* = 3) was associated with the lowest mean MICs for all beta-lactam treatments. Conversely, strains with substitutions in MurG (R335P, *n* = 3), MurD (F76L, *n* = 4), or FtsH (D354G, *n* = 4) had mean amoxicillin/clavulanate and meropenem MICs above 5.0 and 16.0 mg/L, respectively. Higher MICs were also noted for strains with substitutions in Chiz (Y124H, *n* = 12), FtsK (M123T, *n* = 4), PbpB (A217T, *n* = 3) and PonA1 (P631_E632insPPS, *n* = 8), even though no deleterious effects were predicted by PROVEAN for these mutations. Strains with some of these substitutions presented mean meropenem/clavulanate MICs of 2 mg/L, but the effect of this combination was mostly unaffected by the considered core-SNVs.

**TABLE 2:** Core-SNVs in beta-lactamase, transpeptidase, and other relevant cell wall biosynthesis genes and geometric mean MIC for the strains with each considered mutation^*a*^

Function	Locus tag	Gene name	Genomic locus	Mutation	Effect in product	No. of isolates (%)	Sublineage^*b*^	AMX^*c*^	AMX/CLA	MEM	MEM/CLA	Provean score^*d*^
								48.3^*e*^	1.8^*e*^	4.0^*e*^	0.8^*e*^	
Beta-lactamase activity												
	Rv2068c	*blaC*	2326664	G > C	A49G	4 (2.33)	4.1.1.1 (4)	22.6	1.0	1.7	0.6	−2.823
PG synthesis												
	Rv2152c	*murC*	2410831	T > C	H431R	4 (2.33)	4.8 (4)	>64	1.0	2.8	1.0	1.997
	Rv2153c	*murG*	2412348	C > G	R335P	3 (1.74)	4.1.2.1 (3)	>64	5.0	16.0	1.3	−3.321
	Rv2155c	*murD*	2416156	G > A	T80I	41 (23.84)	4.1 (1)	>64	2.8	5.9	1.0	−2.192
							4.1.1.1 (4)					
							4.1.1.3 (10)					
							4.1.2 (3)					
							4.1.2.1 (23)					
			2416167	G > T	F76L	4 (2.33)	4.4.1.1 (4)	>64	5.7	16.0	2.0	−4.473
	Rv2158c	*murE*	2420535	C > A	G25V	50 (29.07)	4.3.4.2 (50)	25.6	0.9	2.5	0.5	−0.922
	Rv2981c	*ddl*	3336825	T > C	T365A	151 (87.79)	All except 4.7 (11) and 4.8 (10)	46.6	1.8	4.1	0.8	3.946
PG assembly												
	Rv0050	*ponA1*	54239	C > G	R193G	6 (3.49)	4.3.4.1 (6)	50.8	1.8	3.2	0.7	−2.144
			55549	GCCGC >TCCGCCGCCGCCGT	P631_E632insPPS	8 (4.65)	2.2.1 (6)	>64	8.0	19.0	1.7	2.692
							2.2.1.1 (1)					
							3 (1)					
	Rv2163c	*pbpB*	2426439	C > T	A217T	3 (1.74)	4.1.2.1 (3)	>64	5.0	16.0	1.3	−2.133
	Rv2911	*dacB2*	3218343	G > A	R2Q	6 (3.49)	4.3.3 (6)	50.8	1.8	3.2	0.6	−0.451
	Rv3682	*ponA2*	4122477	A > G	T188A	3 (1.74)	4.3.4.1 (3)	20.2	0.4	1.3	0.4	−3.521
PG hydrolysis												
	Rv1884c	*rpfC*	2134215	T > C	H16R	86 (50.00)	4.3.3 (6)	31.5	1.1	2.5	0.6	−0.091
							4.3.4.1 (30)					
							4.3.4.2 (50)					
	Rv2190c	*-^*f*^*	2452756	C > A	A173S	16 (9.30)	4.3.4.2 (16)	41.5	1.0	3.1	0.6	−2.883
			2453025	G > A	A83V	7 (4.07)	4.1.1.3 (7)	47.6	0.9	2.4	0.6	−1.920
	Rv3915	*cwlM*	4403900	A > G	M237V	50 (29.07)	4.3.4.2 (50)	25.6	0.9	2.5	0.5	−3.597
Cell division												
	Rv2719c	*chiZ*	3031168	A > G	Y124H	12 (6.98)	2.2.1 (8)	>64	9.5	18.0	1.4	0.863
							2.2.1.1 (2)					
							3 (1)					
							6 (1)					
	Rv2748c	*ftsK*	3061615	T > C	M298V	6 (3.49)	4.3.3 (6)	50.8	1.8	3.2	0.6	−0.703
			3062139	A > G	M123T	4 (2.33)	4.4.1.1 (4)	>64	5.7	16.0	2.0	−1.710
	Rv3610c	*ftsH*	4051823	T > C	D354G	4 (2.33)	4.4.1.1 (4)	>64	5.7	16.0	2.0	−5.429

a Only nonsynonymous mutations present in more than two isolates but not in all strains are shown. The complete data is provided in Table S3.

b The number of strains in each sublineage for the considered SNVs is represented between parentheses.

c AMX, amoxicillin; CLA, clavulanate; MEM, meropenem.

d PROVEAN scores below the −2.5 cutoff were predicted to have a deleterious impact on protein function.

e Global geometric mean MIC for all clinical strains.

f -, not applicable.

**FIG 3:**
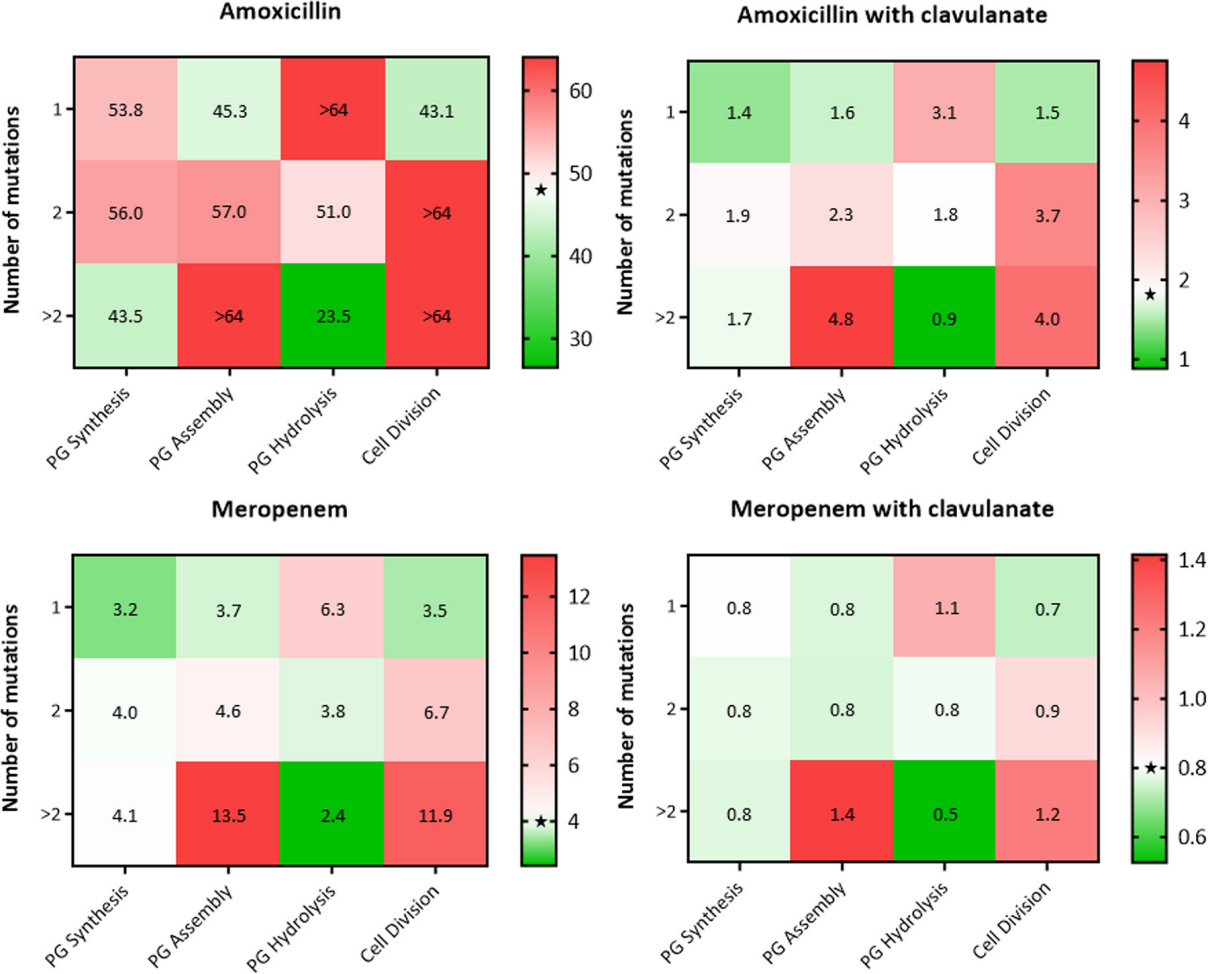
Heatmaps depicting the geometric mean MICs of selected beta-lactam treatments for strains accumulating one, two, or more than two nonsynonymous mutations in genes involved in PG synthesis, PG assembly, PG hydrolysis, or cell division. Heatmaps are colored using a double gradient, with the baseline value in white and adjusted to correspond to the global geometric mean MIC of each treatment (filled star symbol; 48.3 mg/L for amoxicillin; 1.8 mg/L for amoxicillin/clavulanate; 4.0 mg/L for meropenem; 0.8 mg/L for meropenem/clavulanate). For each treatment, cells are colored in shades of green or red according to lower or higher geometric mean MICs of the correspondent strains (value in mg/L for each cell, rounded to one decimal place), compared to the global geometric mean. For each group of genes, the total of strains per cumulative number of mutations is as follows: (i) PG synthesis: one mutation (*n* = 16); two mutations (*n* = 57); more than two mutations (*n* = 99); (ii) PG assembly: one mutation (*n* = 138); two mutations (*n* = 30); more than two mutations (*n* = 4); (iii) PG hydrolysis: one mutation (*n* = 65); two mutations (*n* = 52); more than two mutations (*n* = 55); (iv) cell division: one mutation (*n* = 142); two mutations (*n* = 23); more than two mutations (*n* = 7).

We next sought to verify if the accumulation of nonsynonymous mutations in genes allocated to the defined categories (apart from beta-lactamase activity) could be correlated with an altered geometric mean of MIC values of amoxicillin or meropenem, with and without clavulanate. Compared to the global MIC geometric mean, no relevant tendency was observed for the PG synthesis group, but strains with more than two mutations in PG assembly genes (*n* = 4) consistently presented much higher MIC mean values, surpassing a 2-fold increase for amoxicillin/clavulanate and meropenem (Fig. 3). Strains with two mutations in PG hydrolase genes (*n* = 52) had a MIC mean that was similar to the global values, while strains with only one mutation (*n* = 65) had generally higher MICs and strains with more than two mutations (*n* = 55) had lower MIC mean, close to half the values obtained for all strains. Considering cell division, strains with two or more mutations (*n* = 30) also displayed higher MICs for amoxicillin, amoxicillin/clavulanate, and meropenem. For meropenem/clavulanate, a 1.5-fold increase of the MIC value compared to the global mean was only noted for strains with more than two mutations in cell division genes.

### Putative genomic markers of beta-lactam response at the whole-genome level.

The global statistical association analysis revealed one variant pattern (six individual core-SNVs) significantly associated with higher amoxicillin/clavulanate and meropenem MICs and four patterns (15 individual core-SNVs) linked to lower MICs of biapenem/clavulanate, doripenem/clavulanate and meropenem (Table 3). These patterns included mutations in genes associated with the following functional categories (11): cell wall and cell processes (*eccA2*, *lpqK*, *rpfC*, *rv1987*, and *cut3*); lipid metabolism (*mmaA4* and *papA1*); intermediary metabolism and respiration (*rv0948c* and *hisI*); information pathways (*hsdM* and *pheT*); regulatory proteins (*rv0342* and *pknA*); insertion sequences and phages (*rv1128c*); conserved hypotheticals (*rv2022c, rv0791c, rv3057c*, and *rv3365c*).

**TABLE 3:** Nonsynonymous and intergenic variants identified as putative genomic markers of beta-lactam response in M. tuberculosis

Locus tag	Gene name	Genomic locus	Mutation	Effect in product	No. of isolates^*a*^	AMX/CLA^*b*^	BIA/CLA	DOR/CLA	MEM	Beta-Lactam phenotype^*c*^
Rv0324	-	391853	A > G	T168A	49	7.56E−06 (4.41E−02)	-^*d*^	-	2.20E−08 (7.43E−03)	High
Rv0668-Rv0669c	-	767414	G > A	intergenic	49	7.56E−06 (4.41E−02)	-	-	2.20E−08 (7.43E−03)	High
Rv1128c	-	1252164	T > C	E270G	49	7.56E−06 (4.41E−02)	-	-	2.20E−08 (7.43E−03)	High
Rv1147-Rv1148c	-	1275957	T > C	intergenic	49	7.56E−06 (4.41E−02)	-	-	2.20E−08 (7.43E−03)	High
Rv2756c	*hsdM*	3069167	A > G	L306P	49	7.56E−06 (4.41E−02)	-	-	2.20E−08 (7.43E−03)	High
Rv3884c	*eccA2*	4366195	T > C	E215G	49	7.56E−06 (4.41E−02)	-	-	2.20E−08 (7.43E−03)	High
Rv0399c	*lpqK*	478358	C > T	E67K	51	-	-	-	5.32E−07 (4.51E−02)	Low
Rv1650	*pheT*	1861274	G > A	R506H	54	-	-	-	8.36E−07 (4.51E−02)	Low
Rv0015c	*pknA*	17608	G > C	S385R	61	-	-	-	1.52E−06 (3.24E−02)	Low
Rv0642c	*mmaA4*	736710	T > C	N165S	61	-	-	-	1.52E−06 (3.24E−02)	Low
Rv0948c	-	1057788	T > G	K59T	61	-	-	-	1.52E−06 (3.24E−02)	Low
Rv1606	*hisI*	1805948	C > T	T99I	61	-	-	-	1.52E−06 (3.24E−02)	Low
Rv1884c	*rpfC*	2134215	T > C	H16R	61	-	-	-	1.52E−06 (3.24E−02)	Low
Rv2022c	-	2267372	A > G	V118A	61	-	-	-	1.52E−06 (3.24E−02)	Low
Rv3379c-Rv3380c	-	3794884	G > A	intergenic	61	-	-	-	1.52E−06 (3.24E−02)	Low
Rv0791c	-	885542	G > C	S100C	67	-	6.82E−07 (4.69E−02)	4.03E−06 (3.19E−02)	3.58E−07 (1.70E−02)	Low
Rv1987	-	2231132	G > A	S36N	67	-	6.82E−07 (4.69E−02)	4.03E−06 (3.19E−02)	3.58E−07 (1.70E−02)	Low
Rv3057c	-	3418328	TCG > GCA	D112A	67	-	6.82E−07 (4.69E−02)	4.03E−06 (3.19E−02)	3.58E−07 (1.70E−02)	Low
Rv3365c	-	3776706	C > T	A266T	67	-	6.82E−07 (4.69E−02)	4.03E−06 (3.19E−02)	3.58E−07 (1.70E−02)	Low
Rv3451	*cut3*	3873392	T > G	L259R	67	-	6.82E−07 (4.69E−02)	4.03E−06 (3.19E−02)	3.58E−07 (1.70E−02)	Low
Rv3824c	*papA1*	4293072	G > A	L35F	67	-	6.82E−07 (4.69E−02)	4.03E−06 (3.19E−02)	3.58E−07 (1.70E−02)	Low

a Number of strains with the corresponding mutation within the 140 selected isolates.

b Values correspond to *P* values yielded by the first and second Mann-Whitney U statistical tests, which are shown outside and inside parentheses, respectively. AMX, amoxicillin; BIA, biapenem; CLA, clavulanate; DOR, doripenem; MEM, meropenem.

c Low or high beta-lactam phenotypes were attributed if the strains with the mutations had lower or higher MICs, respectively, than strains without the variants, as expressed by the first test mean ranks.

d -, not available.

## DISCUSSION

The present study consists of one of the most extensive beta-lactam screenings coupled with WGS data performed so far in M. tuberculosis clinical isolates. Strains were particularly susceptible to meropenem/clavulanate and, despite the absence of reference breakpoints for some beta-lactams, similar antibiotic properties together with overlapping MIC distributions imply newer carbapenems, like biapenem and doripenem, are equally effective. Conversely, ertapenem, which exhibits a better therapeutical administration profile than other carbapenems, displayed high MICs, an atypical feature that possibly results from thermal instability (10). Although carbapenems provide the most efficient transpeptidase blocking, results sustain that faropenem is less prone to beta-lactamase degradation (12). Additionally, the improved bioavailability of this penem as an orally active prodrug constitutes a major advantage for therapeutical adhesion compared to the intravenous administration of carbapenems (13). Overall, our results corroborate that clavulanate is essential for the full effect of amoxicillin, while carbapenems, as slow BlaC substrates (3), are more suited to exert their action alone. Nevertheless, the effects of all carbapenems were still potentiated and stabilized by clavulanate in our study.

Our study outputs were constrained by the high proportion of LAM strains which comprise the vast majority circulating in Portugal and one of the predominant sublineages in Europe (14, 15). Although this analysis could have been powered with a larger sample size and more isolates from several distinct lineages, we have performed a proof-of-concept study with a representative collection of Portuguese M. tuberculosis clinical strains that reinforce previous studies reporting worldwide M. tuberculosis clinical isolates' susceptibility to beta-lactams (3, 8, 9). The increased proportion of drug-resistant strains within sublineage 4.3.4.2 reflects the significance of two LAM strain-types as the main promoters of MDR/XDR-TB cases in Portugal (16). Importantly, the screened antimycobacterial drug-resistant M. tuberculosis strains in our study were significantly more susceptible to several beta-lactams. A similar overrepresentation of amoxicillin/clavulanate susceptibility for South African LAM4 strains, especially XDR isolates, was previously reported (8), but unlike Cohen et al. (8), we did not identify significant beta-lactam susceptibility differences between pre-XDR strains and other resistance profiles. Nonetheless, our concordant results with sublineage 4.3.4.2 in the European setting strengthen the notion that specific LAM strains are associated with increased susceptibility to beta-lactams, and it is reasonable to hypothesize that the observed paradoxical beta-lactam susceptibility of drug-resistant isolates possibly stems from fitness cost mutations. Contrarily, Beijing and sublineage 4.1.2.1 strains showed higher resistance to beta-lactams. Moreover, sublineage representation of mutations in cell wall biosynthesis genes associated with globally higher beta-lactam MICs unveiled their concentration in Beijing, 4.1.2.1, and 4.4.1.1 isolates. Recently, lineage 2 was shown to have a significantly higher probability of acquiring resistance than lineage 4 (17). In Beijing strains, this tendency has been attributed to mutations in putative mutator genes (18), which may eventually contribute to the consistently higher beta-lactam MICs obtained for these isolates. Our findings regarding differential sublineage susceptibility to beta-lactams are particularly interesting given the fact that 4.1.2/Haarlem and 4.3/LAM are the most widespread sublineages (15). Thus, determining if the inclusion of beta-lactams in eventual therapeutic schemes against DR-TB may result in better clinical outcomes concerning certain sublineages over other genotypes should be further investigated.

Strains with the A49G substitution in BlaC had considerably lower MICs compared with the global mean. The S111R substitution in BlaC, which was not identified in our strains, was previously associated with increased beta-lactam susceptibility (7), but Li et al. (7) reported that none of the eight polymorphisms found in *blaC*, including the one resulting in S111R, could be linked to beta-lactam resistance (9). This same study also refers that either they did not identify any mutations in the genes encoding beta-lactam targets or that the detected variants did not correlate with significant phenotypic differences. We analyzed a much broader set of relevant genes and found several nonsynonymous mutations, of which the T188A substitution in the transglycosylase penicillin-insensitive domain of PonA2 emerges as a potentially relevant indicator of enhanced beta-lactam susceptibility. Despite possible functional redundancy, genes encoding L,d-transpeptidases were highly conserved. Importantly, we found that cumulative nonsynonymous mutations in PG assembly genes resulted in superior beta-lactam MICs, which may derive from an overall PG transpeptidase content with reduced beta-lactam affinity in these strains. Accumulation of mutations in cell division genes was also associated with higher beta-lactam MICs. Cephalexin, a beta-lactam that inhibits FtsI (a cell-division specific PBP in Escherichia coli), was shown to require the proper assembly of the divisome to ensure rapid lysis at the division site (19). Recently, the molecular structures of M. tuberculosis PBP3 (also known as FtsI or PbpB) in a complex with several beta-lactams, including amoxicillin and meropenem, were solved and revealed the inactivation of this enzyme by these antibiotics through the formation of stable acyl-enzyme complexes (20). Therefore, multiple mutations in cell division genes that compromise the process or timing of the divisome assembly or the interaction between its components, may negatively impact homolog lysis mechanisms induced by FtsI-specific beta-lactams in M. tuberculosis, contributing to the observed high MICs. On the contrary, strains with more than two mutations in PG hydrolases were related to lower beta-lactam MICs, possibly due to detrimental amino acid substitutions that affect the function of these enzymes. This is consistent with previous studies that show that mutants lacking PG hydrolases, such as resuscitation-promoting factors (Rpfs) or Rpf-interacting protein A (RipA), have increased outer membrane permeability and beta-lactam susceptibility (21, 22).

Association tests between phenotypes and core-SNVs were performed to provide a wider outline of putative genomic markers. The identified mutations were distributed across the various sublineages and it is noteworthy that the highest number of core-SNVs with significant associations with higher or lower beta-lactam MICs was still found for the cell wall and cell processes functional category. We observed that the E215G substitution in EccA2, an ESX-2 type VII secretion system component, was associated with higher MICs of amoxicillin/clavulanate and meropenem. This resonates with the recent finding that the V762G substitution in EccC5, a protein involved in another ESX secretion system, grants ofloxacin resistance to M. tuberculosis (23). Conversely, strains with the E67K variant in LpqK, a conserved lipoprotein with similarity to PBPs, had lower beta-lactam MICs. Substitutions in RpfC (H16R) and PknA (N165S) were also associated with increased susceptibility. Depletion of PknA, an essential regulatory kinase of M. tuberculosis peptidoglycan processes, was found to potentiate the activity of beta-lactams (24).

Susceptibility genomic markers were only previously described for amoxicillin/clavulanate (8). We identified variants that differ from this previous study and that are associated with either increased resistance to amoxicillin/clavulanate and meropenem or increased susceptibility to biapenem/clavulanate, doripenem/clavulanate, and meropenem. None of the amoxicillin/clavulanate susceptibility-associated variants identified by Cohen et al. were found in canonical targets, such as L,d-transpeptidases, or PBPs, and evidence of altered beta-lactamase activity was not observed (8). Our findings indicate that mutations in cell wall biosynthesis genes are infrequent and that heightened beta-lactam susceptibility may rely on more intricate genetic patterns, but further studies are needed to support this assumption. Even though strains with more than two mutations in PG assembly or cell division genes exhibited considerably higher beta-lactam MICs, these only accounted for 2% (4/172) and 4% (7/172) of the global sample, respectively. Therefore, strains with these potentially challenging mutational profiles should be studied, but they are unlikely to jeopardize the global benefit that certain beta-lactams can add to TB therapeutics.

Our analysis expands the pool of available putative markers, but studies on the role of the individual or conjugated mutations in beta-lactam phenotype causality are warranted. As mentioned previously, our study would have benefited from the inclusion of strains from lineages that are less frequent in Europe. Additionally, inherent constraints due to strong clonal population stratification complicate phenotype-genotype correlations in M. tuberculosis. In the next step, we will perform genome-wide association studies, which consider these limitations, to better clarify the genomic determinants of the diverse phenotypic responses to specific beta-lactams. This will allow further insights into this class application potential and restrictions and is aligned with the considerations of the WHO that more research is needed on the role of carbapenems in MDR-TB regimens (25).

## MATERIALS AND METHODS

### M. tuberculosis isolates and drug susceptibility testing (DST).

A set of 172 M. tuberculosis clinical isolates curated by the Portuguese National Institute of Health and the reference strain H37Rv were selected for this study (Table S1). DST for 10 antimycobacterial drugs (isoniazid, rifampicin, ethambutol, pyrazinamide, streptomycin, levofloxacin, moxifloxacin, amikacin, kanamycin, ethionamide) was performed following standardized guidelines (26). The sample consisted of 100 pan-susceptible and 72 antimycobacterial drug-resistant isolates, including monoresistant (resistance to one antimycobacterial drug, *n* = 9), polyresistant (resistance to two antimycobacterial drugs, but not isoniazid and rifampicin simultaneously, *n* = 5), MDR (*n* = 44) and pre-XDR (*n* = 14) strains. Resistance to bedaquiline and linezolid is unknown as DST of these antibiotics was not a standard routine practice at the time most of the strains were screened. Strains were grown in Middlebrook 7H9 broth (BD Difco) supplemented with 10% oleic acid-albumin-dextrose-catalase (BD Difco), 0.2% glycerol (Sigma-Aldrich) and 0.05% tyloxapol (Sigma-Aldrich). A broth microdilution assay adaptation was used to determine MICs to amoxicillin, biapenem, cefotaxime, doripenem, ertapenem, faropenem, and meropenem (Sigma-Aldrich), alone or combined with 2.5 mg/L clavulanates (Sigma-Aldrich) (8). After 10 to 12 days of incubation, the lowest concentration leading to no visible growth was recorded as the MIC. Determinations were performed one to three times on each isolate. When available, drug-susceptibility breakpoints used were based on EUCAST guidelines on PK-PD breakpoints (version 12.0) (https://www.eucast.org/clinical_breakpoints/) because beta-lactam critical concentration values for M. tuberculosis were not defined.

### Whole-genome sequencing.

Genomic DNA was extracted as previously described (27). Quantification and quality of the purified DNA were assessed by Qubit Fluorometer (Invitrogen) with the dsDNA HS assay kit (Invitrogen) and agarose gel electrophoresis, respectively. High-quality DNA samples were subjected to dual-indexed NexteraXT Illumina library preparation. Libraries were subsequently subjected to cluster generation and paired-end sequencing (2 × 150bp or 2x250bp) on an Illumina MiSeq or NextSeq550 equipment (Illumina Inc.).

### Core-SNV-based analysis.

Genetic relatedness among isolates was evaluated by a reference-based mapping strategy using Snippy v.4.5.1 software (https://github.com/tseemann/snippy). After species confirmation and contamination screening using Kraken v.2.0.7 (28), quality improved reads by Trimmomatic v0.38 were individually mapped against M. tuberculosis H37Rv reference genome (GenBank accession number AL123456.3) (29). SNV calling was performed on variant sites as previously described (30), with slight changes: minimum mapping quality of 30, and minimum base quality of 20. Core-SNVs were extracted using Snippy’s core module, by masking known M. tuberculosis genomic regions with high GC-content, repetitive elements, and resistance-associated positions to avoid bias in the phylogeny (30). Only genomes with ≥95% of aligned bases with the reference were considered for phylogenetic analysis. Minimum spanning trees (MST) were generated with the MSTreeV2 algorithm in GrapeTree (31), based on a total of 9021 core-SNVs and annotated with supplied metadata. Node collapse was set to a maximum of 12 allelic differences (AD), previously reported as a conservative threshold for epidemiological surveillance of M. tuberculosis transmission chains (32).

### *In silico* lineage determination, spoligotyping, and resistance prediction.

Raw reads of each isolate were subjected to TB-profiler for *in silico* prediction of resistance, lineage, and spoligotype (33).

### Genotype-phenotype association tests.

For each treatment, the 172 strains were divided into three groups according to their MICs and the antibiotic global geometric mean: an intermediary group, spanning strains with a MIC value immediately below and above the geometric mean; low and high MIC groups, respectively, containing all strains with MICs below or above the intermediary group limits. The analyses focused on a set of 140 strains, after randomly selecting two strains from the eight phylogenetic tree nodes (≤12 AD) with more than two members and excluding the remainder to limit phylogenetic dependency. Within the original 9021 core-SNVs, variants with less than 10% (*n* = 14) of strains differing from all others were removed, ensuring an adequate quantity of strains with either wild type (WT) or mutant alleles. This filter reduced available core-SNVs to 325, organized in 44 different core-SNVs patterns across the 140 strains.

The association between log_2_ transformed MICs values and core-SNVs patterns were evaluated for all beta-lactams by a Mann-Whitney U test. Statistical dependence between pairs of core-SNVs with the same allelic distributions across the 140 selected strains was expected due to the genomic proximity of M. tuberculosis isolates. Therefore, core-SNVs with equal allelic configurations were considered a unique variant pattern, which corresponded to an independent statistical hypothesis and yielded the same *P* value for all variants within that core-SNV pattern. To further mitigate phylogenetic dependence, significant core-SNV patterns (*P* < 0.05) were subjected to a second Mann-Whitney U test with selected isolates from the previously defined low and high beta-lactam MIC groups (trees in Supplementary Appendix show the selected strains for the second test for each antibiotic). For each condition, one strain from the low and high MIC groups was initially selected from the central node of the tree and outward from nodes with at least 200 AD from the central node and between each other. When either a low or a high MIC strain was not present in each node, the closest available strain in neighboring nodes was selected. Similar to the first test, a 10% cutoff was set for WT and mutant groups. After exclusion of synonymous mutations, core-SNVs with a *P* < 0.05 in both Mann-Whitney U tests were considered putative genomic markers of low or high beta-lactam resistance phenotype.

### Statistical analysis and data visualization.

Statistical analyses were conducted with SPSS software. PROVEAN software was used to predict the functional effect of a given sequence variation on protein function (34). Heatmaps depicting the MIC geometric mean of strains with a defined number of nonsynonymous mutations in relevant gene groups were generated using GraphPad Prism version 9.0.

### Data availability.

Sequence files have been deposited in the European Nucleotide Archive. Accession numbers are available in Table S1.

## References

[1] Catalão MJ, Filipe SR, Pimentel M. 2019. Revisiting anti-tuberculosis therapeutic strategies that target the peptidoglycan structure and synthesis. Front Microbiol 10:190. doi:10.3389/fmicb.2019.00190. 30804921 PMC6378297

[2] Story-Roller E, Lamichhane G. 2018. Have we realized the full potential of β-lactams for treating drug-resistant TB? IUBMB Life 70:881–888. doi:10.1002/iub.1875. 29934998 PMC6119476

[3] Hugonnet JE, Tremblay LW, Boshoff HI, Barry C, Blanchard J. 2009. Meropenem-clavulanate is effective against extensively drug-resistant *Mycobacterium tuberculosis*. Science 323:1215–1218. doi:10.1126/science.1167498. 19251630 PMC2679150

[4] Gupta R, Lavollay M, Mainardi JL, Arthur M, Bishai W, Lamichhane G. 2010. The *Mycobacterium tuberculosis* protein Ldt_Mt2_ is a nonclassical transpeptidase required for virulence and resistance to amoxicillin. Nat Med 16:466–469. doi:10.1038/nm.2120. 20305661 PMC2851841

[5] Gonzalo X, Drobniewski F. 2013. Is there a place for β-lactams in the treatment of multidrug-resistant/extensively drug-resistant tuberculosis? Synergy between meropenem and amoxicillin/clavulanate. J Antimicrob Chemother 68:366–369. doi:10.1093/jac/dks395. 23070734

[6] Solapure S, Dinesh N, Shandil R, Ramachandran V, Sharma S, Bhattacharjee D, Ganguly S, Reddy J, Ahuja V, Panduga V, Parab M, Vishwas KG, Kumar N, Balganesh M, Balasubramanian V. 2013. *In vitro* and *in vivo* efficacy of β-lactams against replicating and slowly growing/nonreplicating *Mycobacterium tuberculosis*. Antimicrob Agents Chemother 57:2506–2510. doi:10.1128/AAC.00023-13. 23507276 PMC3716166

[7] Zhang D, Wang Y, Lu J, Pang Y. 2016. *In vitro* activity of β-lactams in combination with β-lactamase inhibitors against multidrug-resistant *Mycobacterium tuberculosis* isolates. Antimicrob Agents Chemother 60:393–399. doi:10.1128/AAC.01035-15. 26525785 PMC4704149

[8] Cohen KA, El-Hay T, Wyres KL, Weissbrod O, Munsamy V, Yanover C, Aharonov R, Shaham O, Conway TC, Goldschmidt Y, Bishai WR, Pym AS. 2016. Paradoxical hypersusceptibility of drug-resistant *Mycobacterium tuberculosis* to β-lactam antibiotics. EBioMedicine 9:170–179. doi:10.1016/j.ebiom.2016.05.041. 27333036 PMC4972527

[9] Li F, Wan L, Xiao T, Liu H, Jiang Y, Zhao X, Wang R, Wan K. 2018. *In vitro* activity of β-lactams in combination with β-lactamase inhibitors against *Mycobacterium tuberculosis* clinical isolates. Biomed Res Int 2018:3579832. doi:10.1155/2018/3579832. 30065936 PMC6051288

[10] Gonzalo X, Satta G, Ortiz Canseco J, McHugh TD, Drobniewski F. 2020. Ertapenem and faropenem against *Mycobacterium tuberculosis: in vitro* testing and comparison by macro and microdilution. BMC Microbiol 20:271. doi:10.1186/s12866-020-01954-w. 32867678 PMC7457350

[11] Kapopoulou A, Lew JM, Cole ST. 2011. The MycoBrowser portal: a comprehensive and manually annotated resource for mycobacterial genomes. Tuberculosis (Edinb) 91:8–13. doi:10.1016/j.tube.2010.09.006. 20980200

[12] Schurek KN, Wiebe R, Karlowsky JA, Rubinstein E, Hoban DJ, Zhanel GG. 2007. Faropenem: review of a new oral penem. Expert Rev Anti Infect Ther 5:185–198. doi:10.1586/14787210.5.2.185. 17402834

[13] Srivastava S, Deshpande D, Pasipanodya J, Nuermberger E, Swaminathan S, Gumbo T. 2016. Optimal clinical doses of faropenem, linezolid, and moxifloxacin in children with disseminated tuberculosis: goldilocks. Clin Infect Dis 63:S102–S109. doi:10.1093/cid/ciw483. 27742641 PMC5064158

[14] Perdigão J, Silva C, Diniz J, Pereira C, Machado D, Ramos J, Silva H, Abilleira F, Brum C, Reis AJ, Macedo M, Scaini JL, Silva AB, Esteves L, Macedo R, Maltez F, Clemente S, Coelho E, Viegas S, Rabna P, Rodrigues A, Taveira N, Jordao L, Kritski A, Lapa E Silva JR, Mokrousov I, Couvin D, Rastogi N, Couto I, Pain A, McNerney R, Clark TG, von Groll A, Dalla-Costa ER, Rossetti ML, Silva PEA, Viveiros M, Portugal I. 2019. Clonal expansion across the seas as seen through CPLP-TB database: a joint effort in cataloguing *Mycobacterium tuberculosis* genetic diversity in Portuguese-speaking countries. Infect Genet Evol 72:44–58. doi:10.1016/j.meegid.2018.03.011. 29559379 PMC6598853

[15] Stucki D, Brites D, Jeljeli L, Coscolla M, Liu Q, Trauner A, Fenner L, Rutaihwa L, Borrell S, Luo T, Gao Q, Kato-Maeda M, Ballif M, Egger M, Macedo R, Mardassi H, Moreno M, Tudo Vilanova G, Fyfe J, Globan M, Thomas J, Jamieson F, Guthrie JL, Asante-Poku A, Yeboah-Manu D, Wampande E, Ssengooba W, Joloba M, Henry Boom W, Basu I, Bower J, Saraiva M, Vaconcellos SEG, Suffys P, Koch A, Wilkinson R, Gail-Bekker L, Malla B, Ley SD, Beck HP, de Jong BC, Toit K, Sanchez-Padilla E, Bonnet M, Gil-Brusola A, Frank M, Penlap Beng VN, Eisenach K, Alani I, Wangui Ndung'u P. 2016. *Mycobacterium tuberculosis* lineage 4 comprises globally distributed and geographically restricted sublineages. Nat Genet 48:1535–1543. doi:10.1038/ng.3704. 27798628 PMC5238942

[16] Perdigão J, Gomes P, Miranda A, Maltez F, Machado D, Silva C, Phelan JE, Brum L, Campino S, Couto I, Viveiros M, Clark TG, Portugal I. 2020. Using genomics to understand the origin and dispersion of multidrug and extensively drug resistant tuberculosis in Portugal. Sci Rep 10:2600. doi:10.1038/s41598-020-59558-3. 32054988 PMC7018963

[17] Torres Ortiz A, Coronel J, Vidal JR, Bonilla C, Moore DAJ, Gilman RH, Balloux F, Kon OM, Didelot X, Grandjean L. 2021. Genomic signatures of pre-resistance in *Mycobacterium tuberculosis*. Nat Commun 12:7312. doi:10.1038/s41467-021-27616-7. 34911948 PMC8674244

[18] Ebrahimi-Rad M, Bifani P, Martin C, Kremer K, Samper S, Rauzier J, Kreiswirth B, Blazquez J, Jouan M, van Soolingen D, Gicquel B. 2003. Mutations in putative mutator genes of *Mycobacterium tuberculosis* strains of the W-Beijing family. Emerg Infect Dis 9:838–845. doi:10.3201/eid0907.020803. 12890325 PMC3023437

[19] Chung HS, Yao Z, Goehring NW, Kishony R, Beckwith J, Kahne D. 2009. Rapid beta-lactam-induced lysis requires successful assembly of the cell division machinery. Proc Natl Acad Sci USA 106:21872–21877. doi:10.1073/pnas.0911674106. 19995973 PMC2799840

[20] Lu Z, Wang H, Zhang A, Liu X, Zhou W, Yang C, Guddat L, Yang H, Schofield CJ, Rao Z. 2020. Structures of *Mycobacterium tuberculosis* penicillin-binding protein 3 in complex with five β-lactam antibiotics reveal mechanism of inactivation. Mol Pharmacol 97:287–294. doi:10.1124/mol.119.118042. 32086254

[21] Wivagg CN, Hung DT. 2012. Resuscitation-promoting factors are required for β-lactam tolerance and the permeability barrier in *Mycobacterium tuberculosis*. Antimicrob Agents Chemother 56:1591–1594. doi:10.1128/AAC.06027-11. 22155826 PMC3294900

[22] Hett CE, Chao MC, Deng LL, Rubin EJ. 2008. A mycobacterial enzyme essential for cell division synergizes with resuscitation-promoting factor. PLoS Pathog 4:e1000001. doi:10.1371/journal.ppat.1000001. 18463693 PMC2262848

[23] Eilertson B, Maruri F, Blackman A, Guo Y, Herrera M, van der Heijden Y, Shyr Y, Sterling TR. 2016. A novel resistance mutation in *eccC5* of the ESX-5 secretion system confers ofloxacin resistance in *Mycobacterium tuberculosis*. J Antimicrob Chemother 71:2419–2427. doi:10.1093/jac/dkw168. 27261264 PMC4992850

[24] Zeng J, Platig J, Cheng TY, Ahmed S, Skaf Y, Potluri LP, Schwartz D, Steen H, Moody DB, Husson RN. 2020. Protein kinases PknA and PknB independently and coordinately regulate essential *Mycobacterium tuberculosis* physiologies and antimicrobial susceptibility. PLoS Pathog 16:e1008452. doi:10.1371/journal.ppat.1008452. 32255801 PMC7164672

[25] World Health Organization. 2020. WHO consolidated guidelines on tuberculosis. Module 4: treatment - drug-resistant tuberculosis treatment. World Health Organization, Geneva. 32603040

[26] Clinical and Laboratory Standards Institute. 2011. Susceptibility testing of mycobacteria, nocardiae, and other aerobic actinomycetes; approved standard, 2nd ed CLSI document M24-A2. Clinical and Laboratory Standards Institute, Wayne, PA. 31339680

[27] Somerville W, Thibert L, Schwartzman K, Behr MA. 2005. Extraction of *Mycobacterium tuberculosis* DNA: a question of containment. J Clin Microbiol 43:2996–2997. doi:10.1128/JCM.43.6.2996-2997.2005. 15956443 PMC1151963

[28] Wood DE, Salzberg SL. 2014. Kraken: ultrafast metagenomic sequence classification using exact alignments. Genome Biol 15:R46. doi:10.1186/gb-2014-15-3-r46. 24580807 PMC4053813

[29] Bolger AM, Lohse M, Usadel B. 2014. Trimmomatic: a flexible trimmer for Illumina sequence data. Bioinformatics 30:2114–2120. doi:10.1093/bioinformatics/btu170. 24695404 PMC4103590

[30] Macedo R, Pinto M, Borges V, Nunes A, Oliveira O, Portugal I, Duarte R, Gomes JP. 2019. Evaluation of a gene-by-gene approach for prospective whole-genome sequencing-based surveillance of multidrug resistant *Mycobacterium tuberculosis*. Tuberculosis (Edinb) 115:81–88. doi:10.1016/j.tube.2019.02.006. 30948181

[31] Zhou Z, Alikhan NF, Sergeant MJ, Luhmann N, Vaz C, Francisco AP, Carriço JA, Achtman M. 2018. Grapetree: visualization of core genomic relationships among 100,000 bacterial pathogens. Genome Res 28:1395–1404. doi:10.1101/gr.232397.117. 30049790 PMC6120633

[32] Walker TM, Ip CL, Harrell RH, Evans JT, Kapatai G, Dedicoat MJ, Eyre DW, Wilson DJ, Hawkey PM, Crook DW, Parkhill J, Harris D, Walker AS, Bowden R, Monk P, Smith EG, Peto TE. 2013. Whole-genome sequencing to delineate *Mycobacterium tuberculosis* outbreaks: a retrospective observational study. Lancet Infect Dis 13:137–146. doi:10.1016/S1473-3099(12)70277-3. 23158499 PMC3556524

[33] Phelan JE, O'Sullivan DM, Machado D, Ramos J, Oppong YEA, Campino S, O'Grady J, McNerney R, Hibberd ML, Viveiros M, Huggett JF, Clark TG. 2019. Integrating informatics tools and portable sequencing technology for rapid detection of resistance to anti-tuberculous drugs. Genome Med 11:41. doi:10.1186/s13073-019-0650-x. 31234910 PMC6591855

[34] Choi Y, Chan AP. 2015. PROVEAN web server: a tool to predict the functional effect of amino acid substitutions and indels. Bioinformatics 31:2745–2747. doi:10.1093/bioinformatics/btv195. 25851949 PMC4528627

