# Supplementary material for: Uncovering Beta-Lactam Susceptibility Patterns in Clinical Isolates of Mycobacterium tuberculosis through Whole-Genome Sequencing
Source: Microbiol Spectr. 2022 Jun 13;10(4):e00674-22. doi: 10.1128/spectrum.00674-22 (PMC9431576; doi:10.1128/spectrum.00674-22)
Supplement: Supplemental file 1 — Supplemental material. Download spectrum.00674-22-s0001.pdf, PDF file, 2.5 MB [file spectrum.00674-22-s0001.pdf]

Supplementary Appendix

Trees in figures a-n show the selected strains for the second test for each antibiotic and the numbers displayed over each branch represent allelic differences.

a

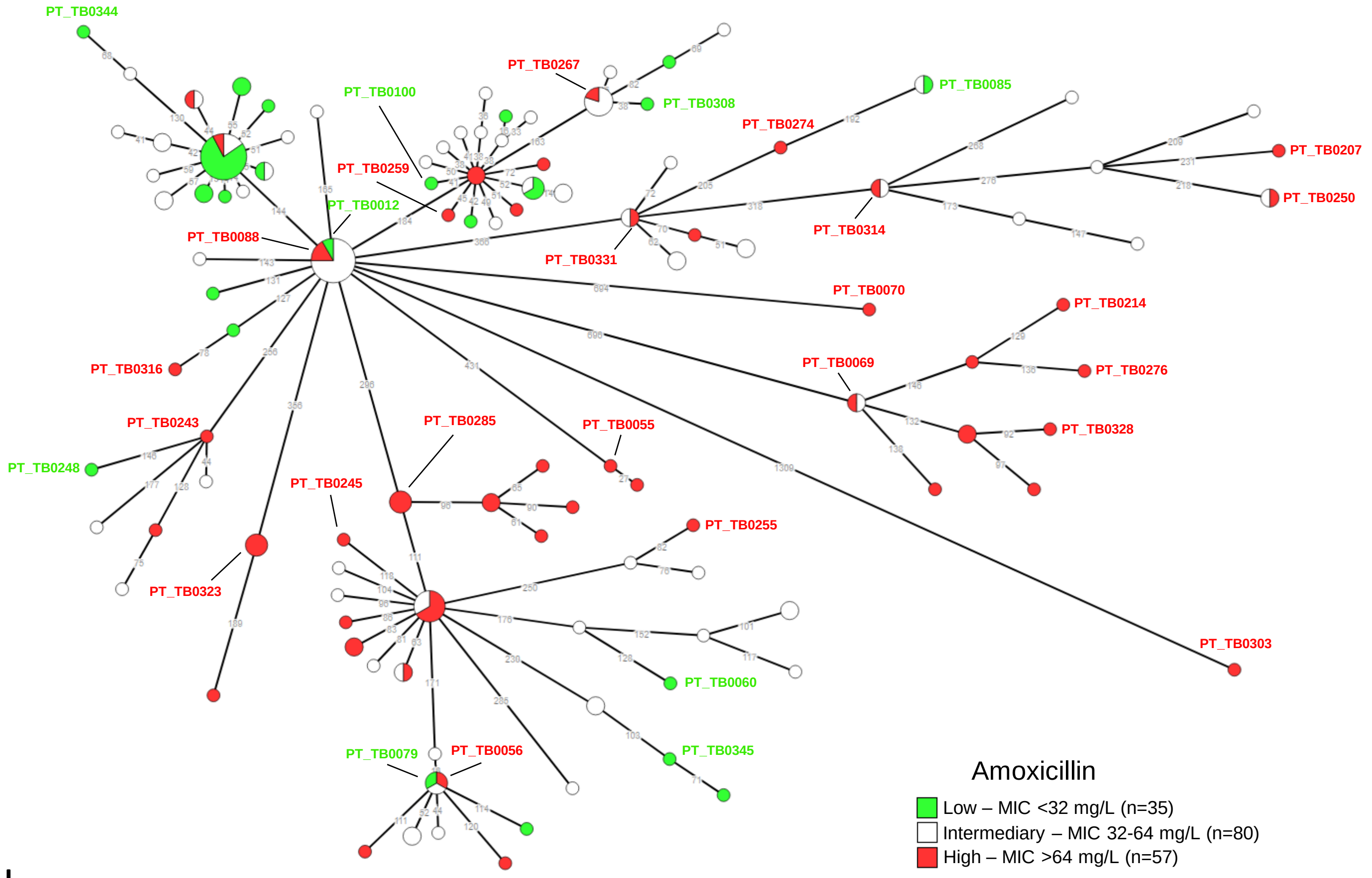

b

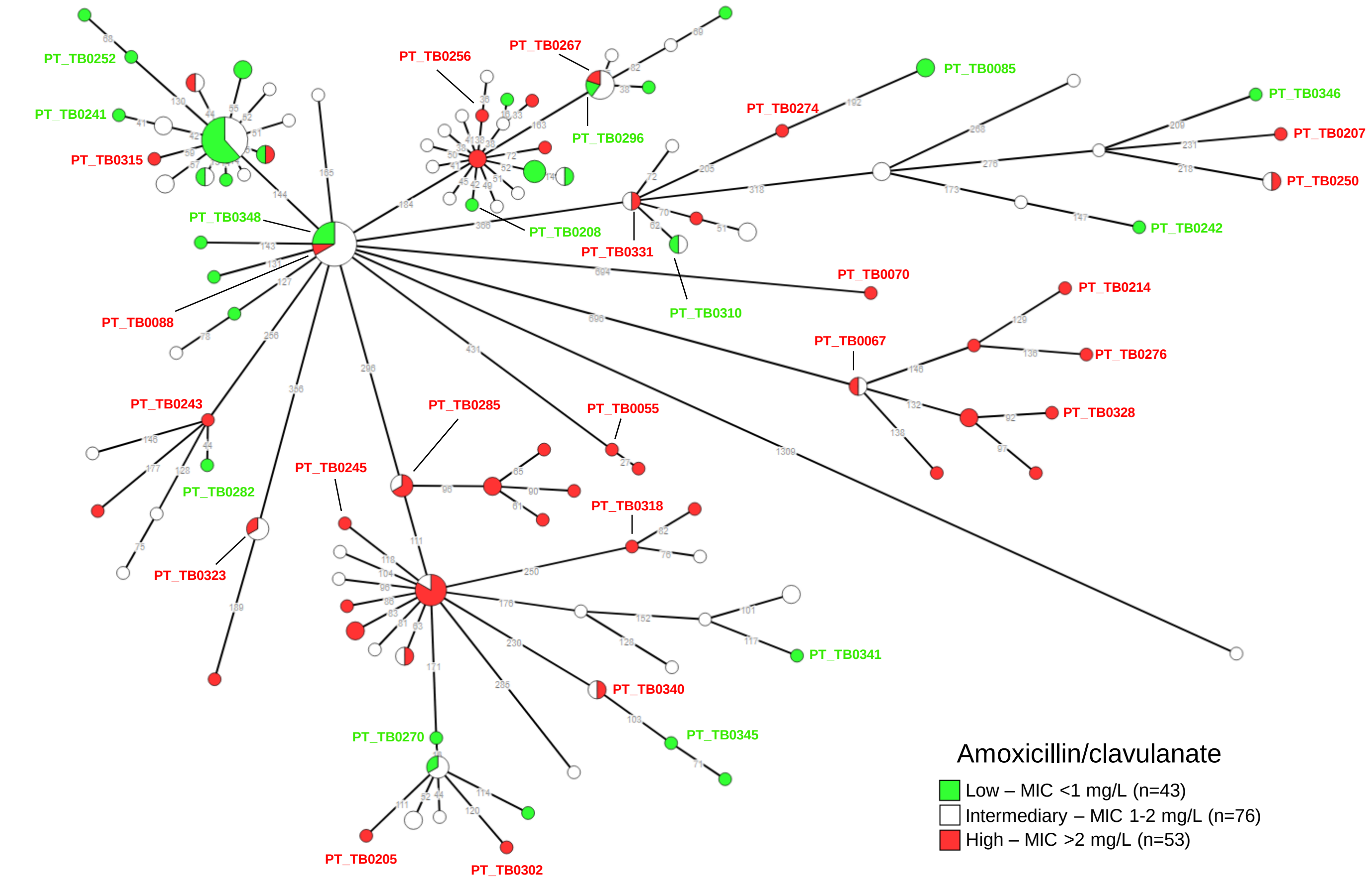

c

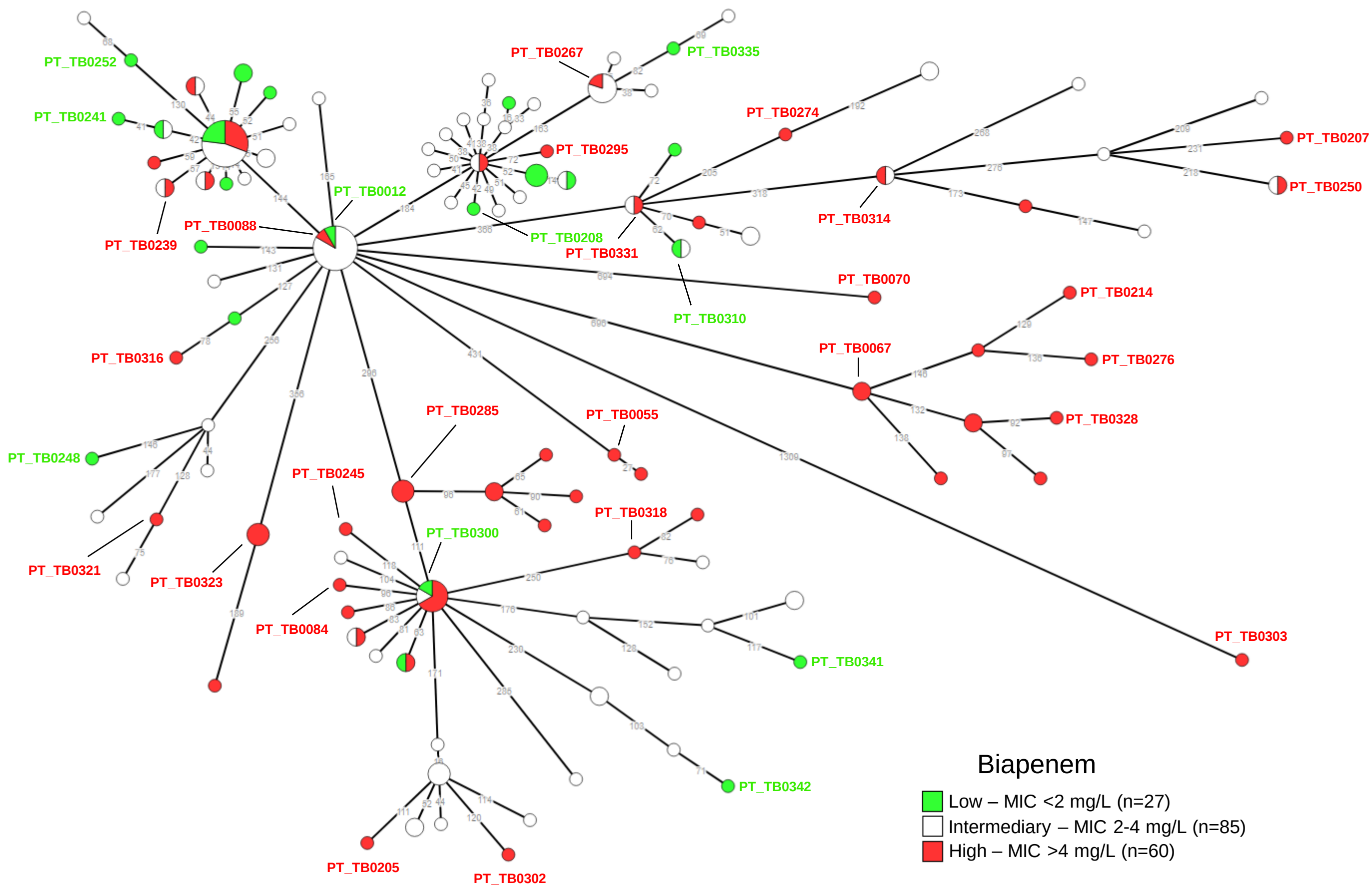
PT_TB0252
PT_TB0241
PT_TB0239
PT_TB0088
PT_TB0012
PT_TB0267
PT_TB0335
PT_TB0295
PT_TB0208
PT_TB0331
PT_TB0274
PT_TB0314
PT_TB0207
PT_TB0250
PT_TB0310
PT_TB0070
PT_TB0316
PT_TB0214
PT_TB0067
PT_TB0276
PT_TB0328
PT_TB0248
PT_TB0285
PT_TB0055
PT_TB0245
PT_TB0318
PT_TB0300
PT_TB0321
PT_TB0323
PT_TB0084
PT_TB0303
PT_TB0341
PT_TB0342
PT_TB0205
PT_TB0302
Biapenem
Low – MIC <2 mg/L (n=27)
Intermediary – MIC 2-4 mg/L (n=85)
High – MIC >4 mg/L (n=60)

d

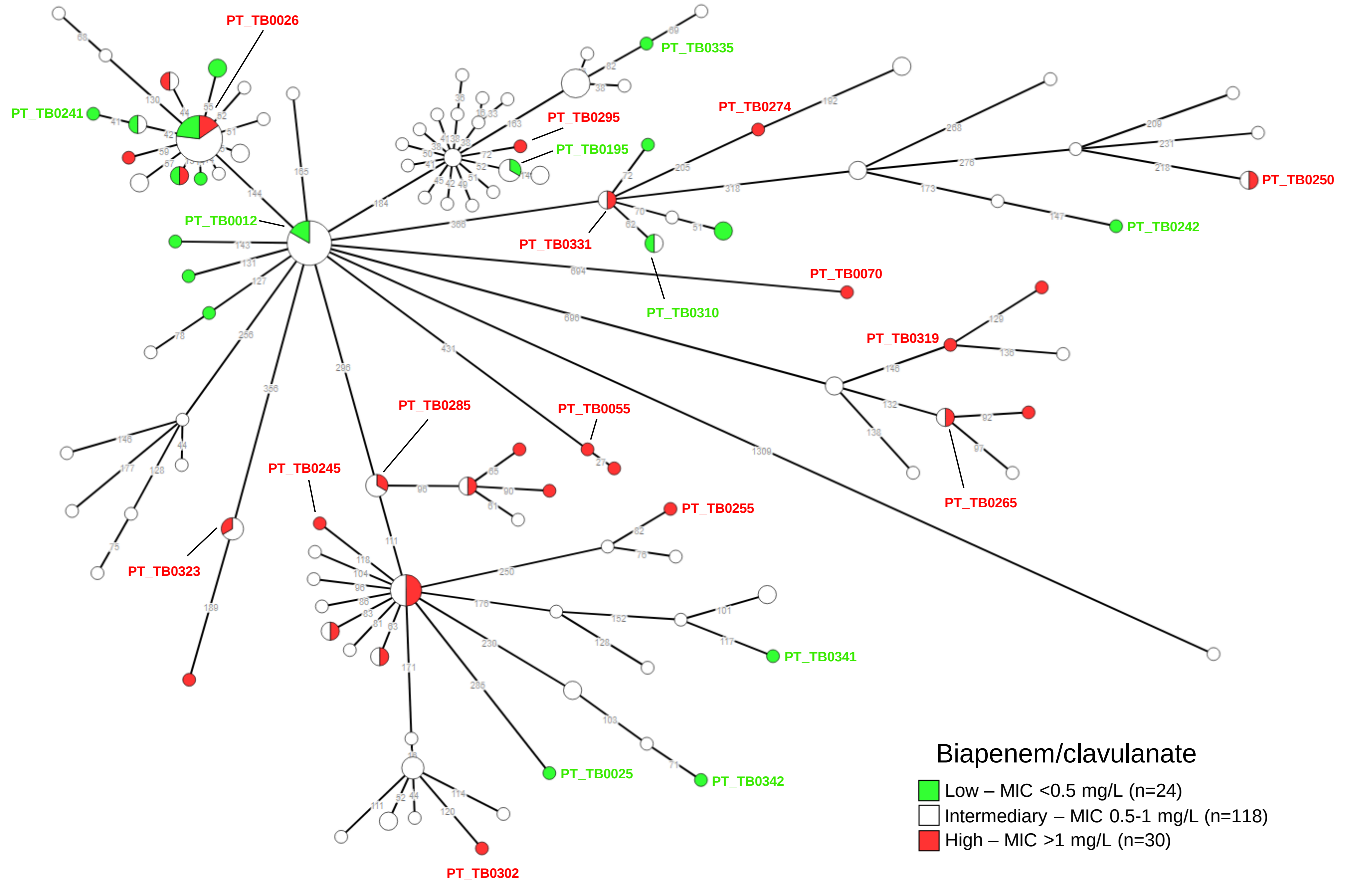
PT_TB0026
PT_TB0335
PT_TB0241
PT_TB0295
PT_TB0195
PT_TB0274
PT_TB0012
PT_TB0331
PT_TB0250
PT_TB0242
PT_TB0310
PT_TB0070
PT_TB0319
PT_TB0285
PT_TB0055
PT_TB0245
PT_TB0265
PT_TB0255
PT_TB0323
PT_TB0341
PT_TB0025
PT_TB0342
PT_TB0302
Biapenem/clavulanate
Low – MIC <0.5 mg/L (n=24)
Intermediary – MIC 0.5-1 mg/L (n=118)
High – MIC >1 mg/L (n=30)

e

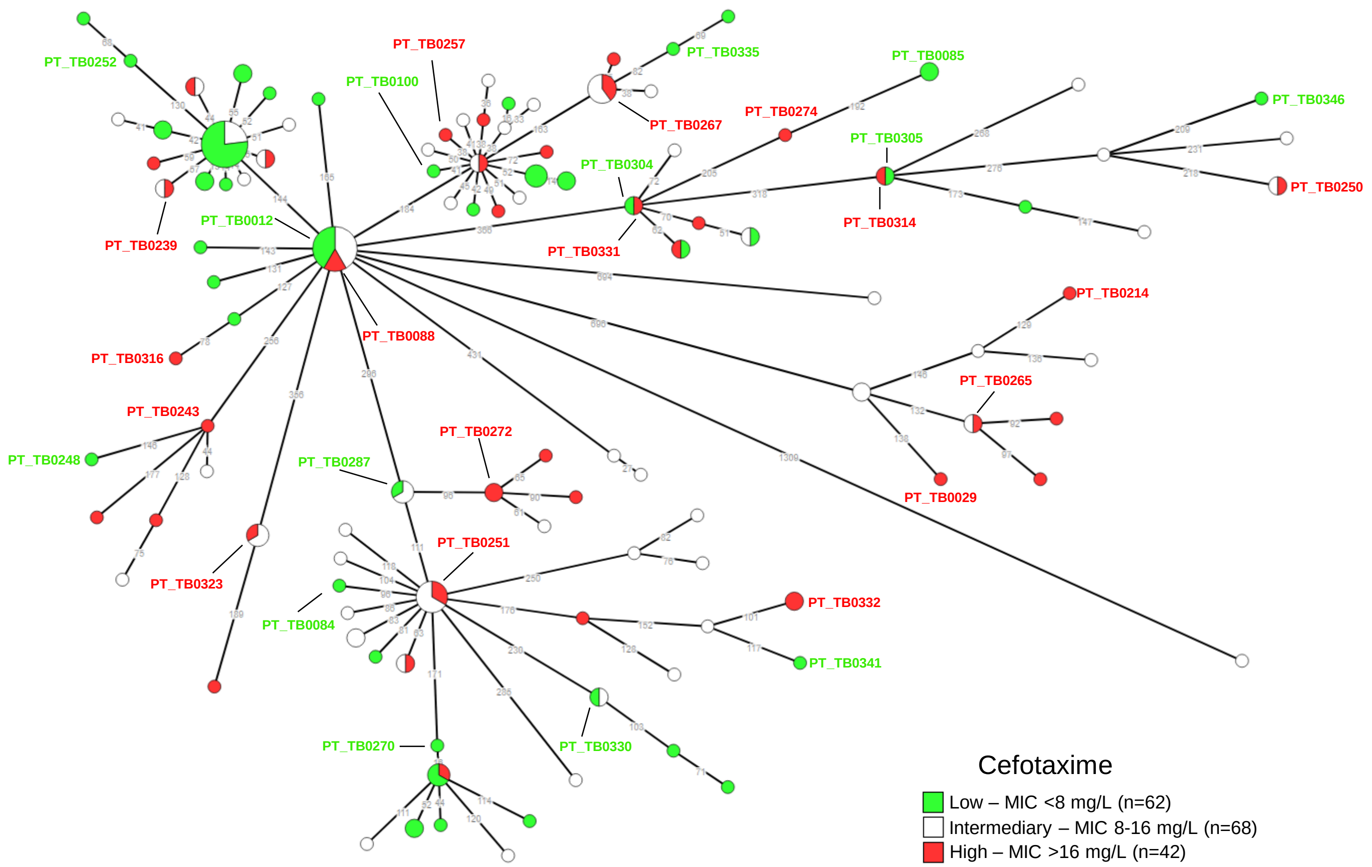
PT_TB0252
PT_TB0257
PT_TB0100
PT_TB0335
PT_TB0085
PT_TB0267
PT_TB0274
PT_TB0346
PT_TB0305
PT_TB0304
PT_TB0250
PT_TB0012
PT_TB0239
PT_TB0314
PT_TB0331
PT_TB0214
PT_TB0316
PT_TB0088
PT_TB0265
PT_TB0243
PT_TB0272
PT_TB0248
PT_TB0287
PT_TB0029
PT_TB0251
PT_TB0323
PT_TB0332
PT_TB0084
PT_TB0341
PT_TB0270
PT_TB0330
Cefotaxime
Low – MIC <8 mg/L (n=62)
Intermediary – MIC 8-16 mg/L (n=68)
High – MIC >16 mg/L (n=42)

f

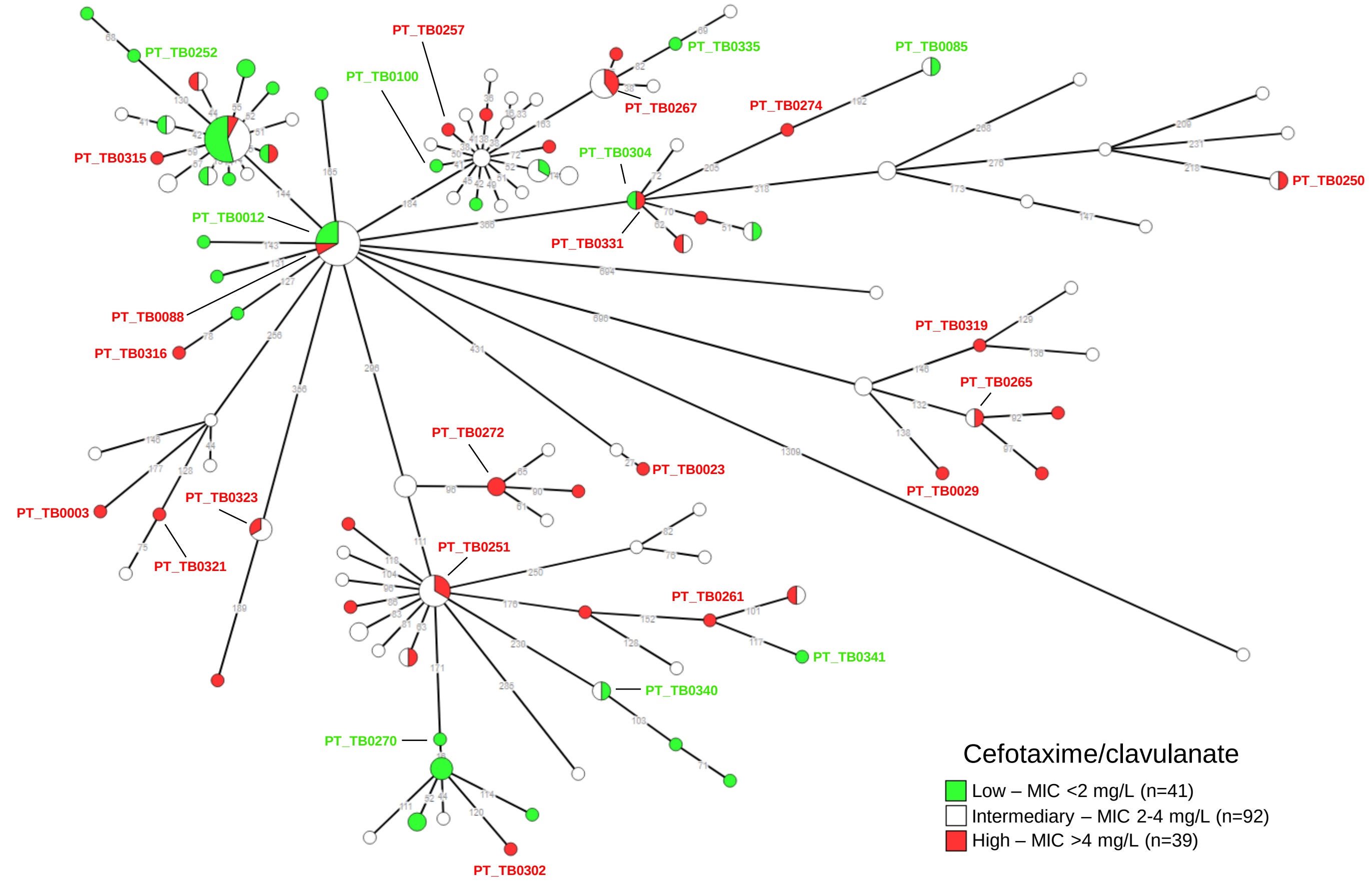
PT_TB0252
PT_TB0257
PT_TB0335
PT_TB0085
PT_TB0100
PT_TB0267
PT_TB0274
PT_TB0315
PT_TB0304
PT_TB0250
PT_TB0012
PT_TB0331
PT_TB0088
PT_TB0319
PT_TB0316
PT_TB0265
PT_TB0272
PT_TB0023
PT_TB0029
PT_TB0323
PT_TB0003
PT_TB0321
PT_TB0251
PT_TB0261
PT_TB0341
PT_TB0340
PT_TB0270
PT_TB0302
Cefotaxime/clavulanate
Low – MIC <2 mg/L (n=41)
Intermediary – MIC 2-4 mg/L (n=92)
High – MIC >4 mg/L (n=39)

g

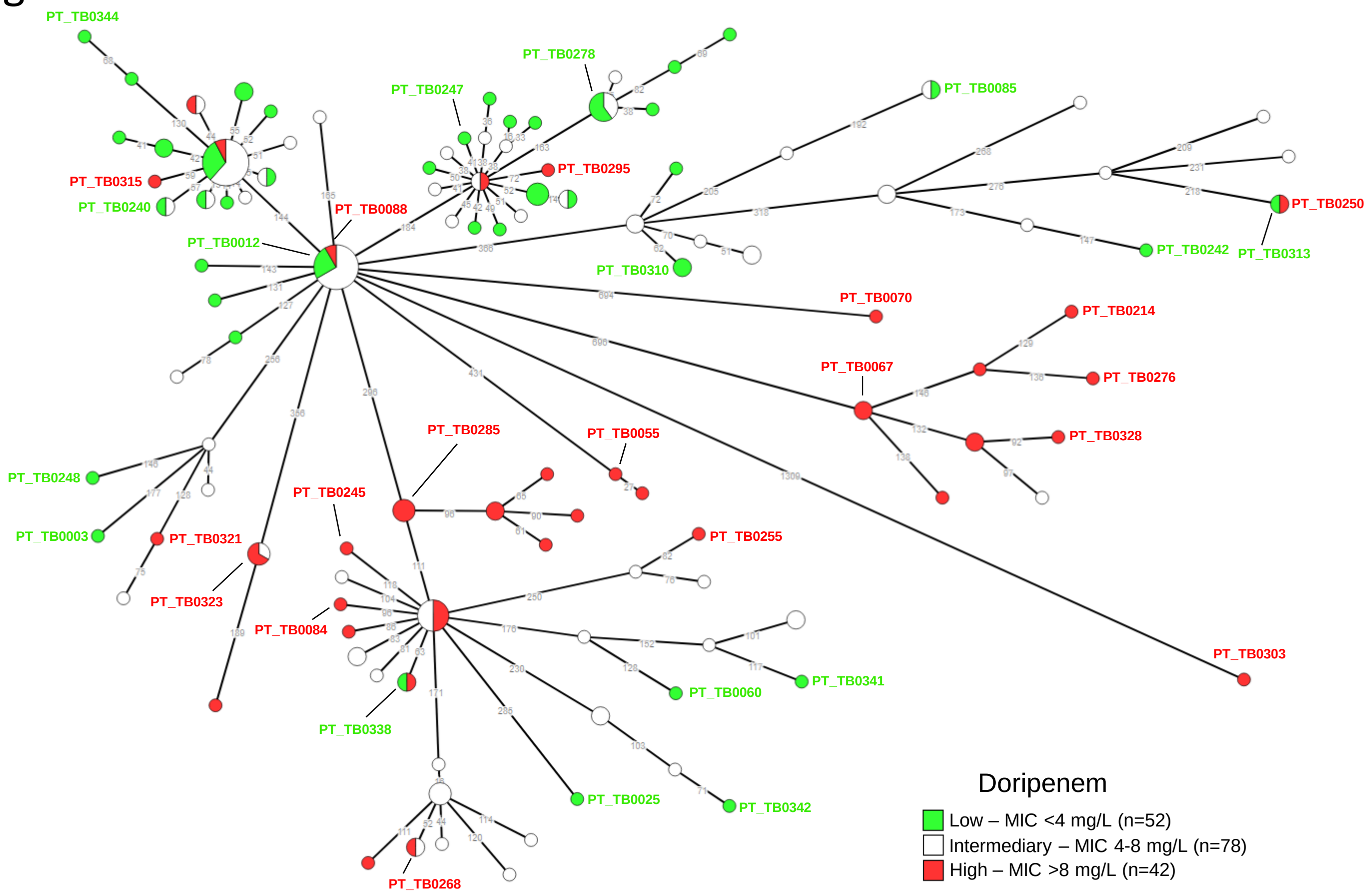
PT_TB0344
PT_TB0278
PT_TB0247
PT_TB0085
PT_TB0315
PT_TB0295
PT_TB0240
PT_TB0088
PT_TB0250
PT_TB0012
PT_TB0242
PT_TB0313
PT_TB0310
PT_TB0070
PT_TB0214
PT_TB0067
PT_TB0276
PT_TB0285
PT_TB0055
PT_TB0328
PT_TB0248
PT_TB0245
PT_TB0003
PT_TB0321
PT_TB0255
PT_TB0323
PT_TB0084
PT_TB0341
PT_TB0060
PT_TB0303
PT_TB0338
PT_TB0025
PT_TB0342
PT_TB0268
Doripenem
Low – MIC <4 mg/L (n=52)
Intermediary – MIC 4-8 mg/L (n=78)
High – MIC >8 mg/L (n=42)

h

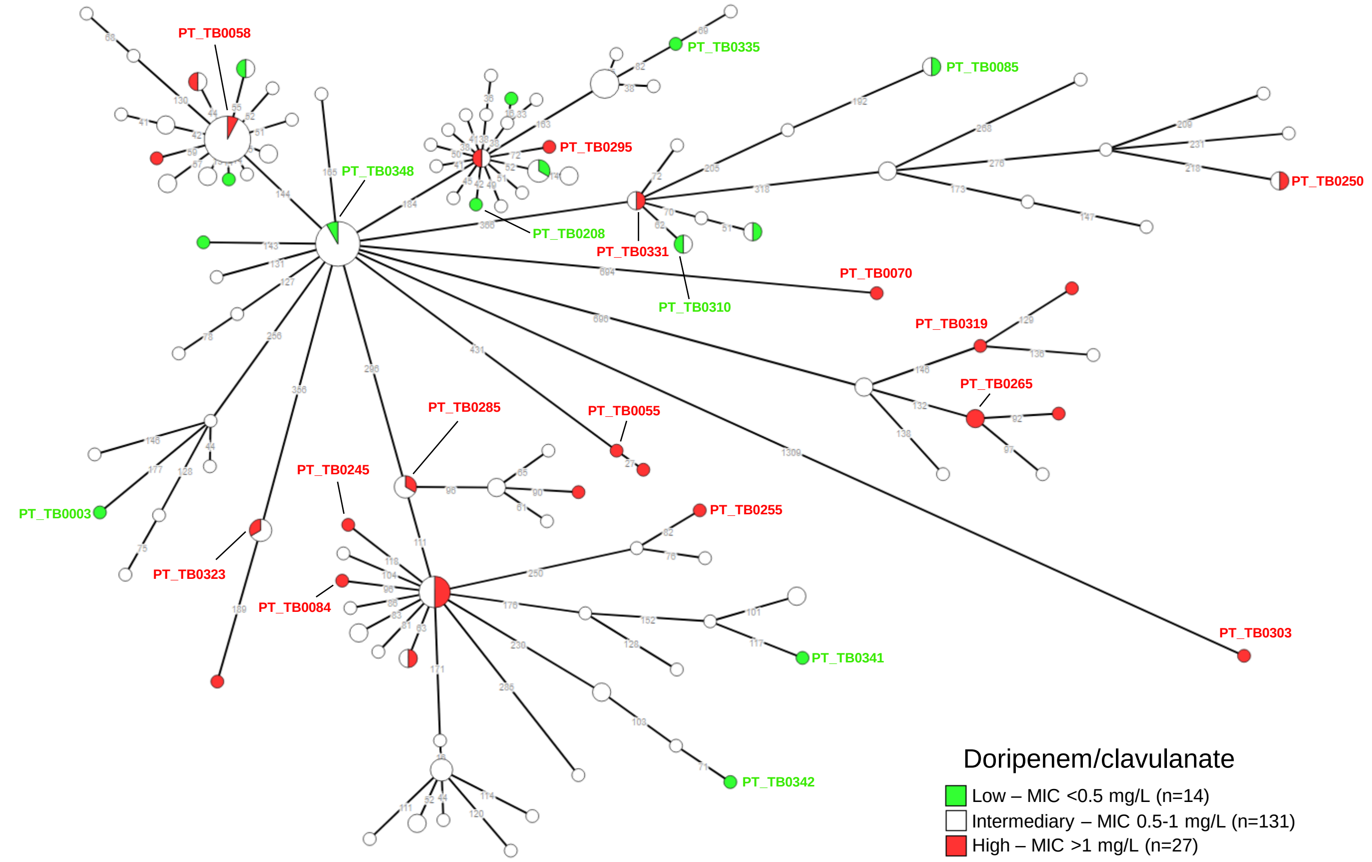
PT_TB0058
PT_TB0335
PT_TB0085
PT_TB0295
PT_TB0348
PT_TB0250
PT_TB0208
PT_TB0331
PT_TB0310
PT_TB0070
PT_TB0319
PT_TB0265
PT_TB0285
PT_TB0055
PT_TB0245
PT_TB0003
PT_TB0255
PT_TB0323
PT_TB0084
PT_TB0303
PT_TB0341
PT_TB0342
Doripenem/clavulanate
Low – MIC <0.5 mg/L (n=14)
Intermediary – MIC 0.5-1 mg/L (n=131)
High – MIC >1 mg/L (n=27)

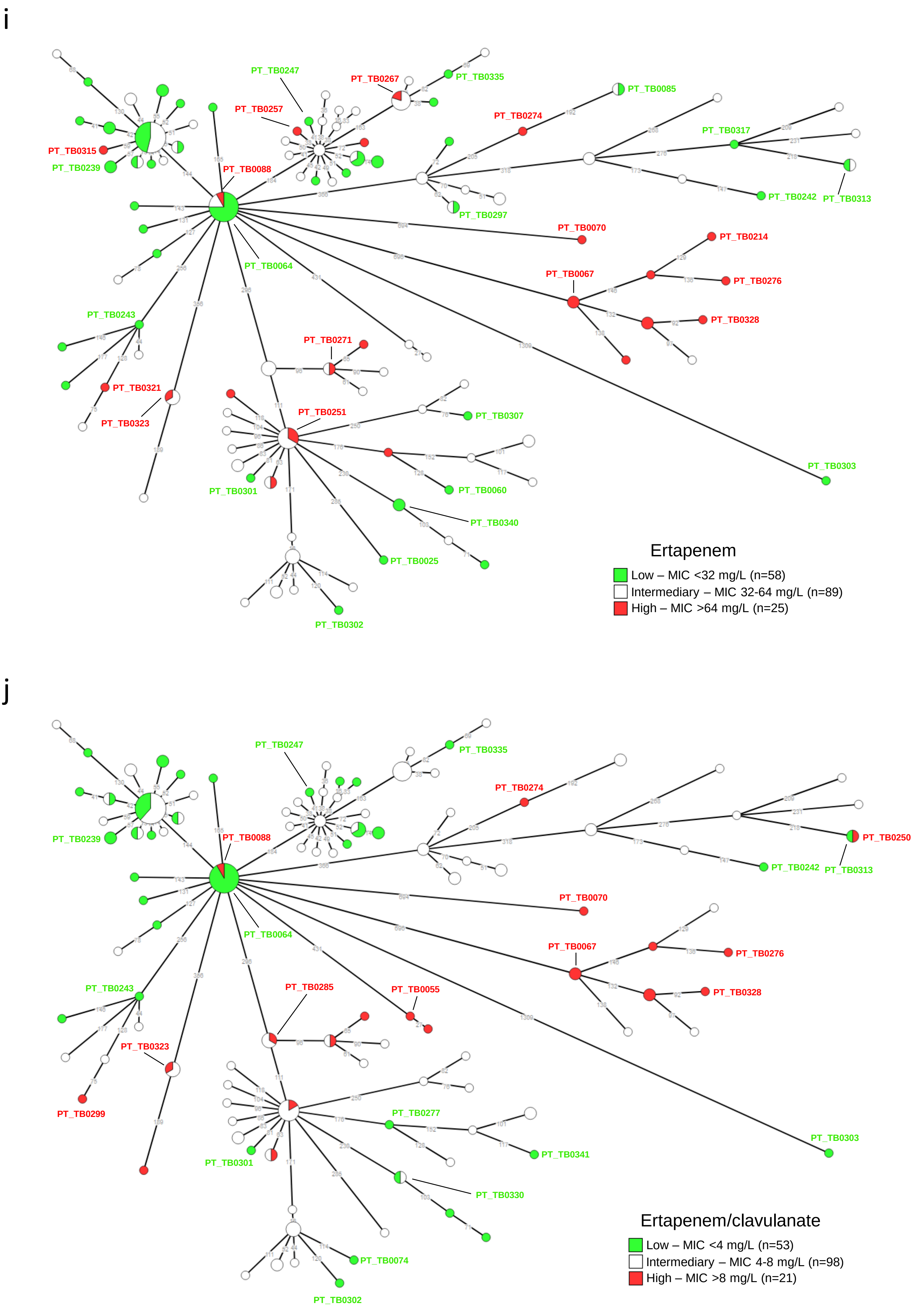

i
PT_TB0247
PT_TB0267
PT_TB0335
PT_TB0085
PT_TB0257
PT_TB0274
PT_TB0317
PT_TB0315
PT_TB0239
PT_TB0088
PT_TB0242
PT_TB0313
PT_TB0297
PT_TB0070
PT_TB0214
PT_TB0064
PT_TB0067
PT_TB0276
PT_TB0243
PT_TB0328
PT_TB0271
PT_TB0321
PT_TB0323
PT_TB0251
PT_TB0307
PT_TB0303
PT_TB0301
PT_TB0060
PT_TB0340
PT_TB0025
PT_TB0302
Ertapenem
Low – MIC <32 mg/L (n=58)
Intermediary – MIC 32-64 mg/L (n=89)
High – MIC >64 mg/L (n=25)
j
PT_TB0247
PT_TB0335
PT_TB0274
PT_TB0239
PT_TB0088
PT_TB0250
PT_TB0242
PT_TB0313
PT_TB0070
PT_TB0064
PT_TB0067
PT_TB0276
PT_TB0243
PT_TB0285
PT_TB0055
PT_TB0328
PT_TB0323
PT_TB0299
PT_TB0277
PT_TB0303
PT_TB0341
PT_TB0301
PT_TB0330
PT_TB0074
PT_TB0302
Ertapenem/clavulanate
Low – MIC <4 mg/L (n=53)
Intermediary – MIC 4-8 mg/L (n=98)
High – MIC >8 mg/L (n=21)

k

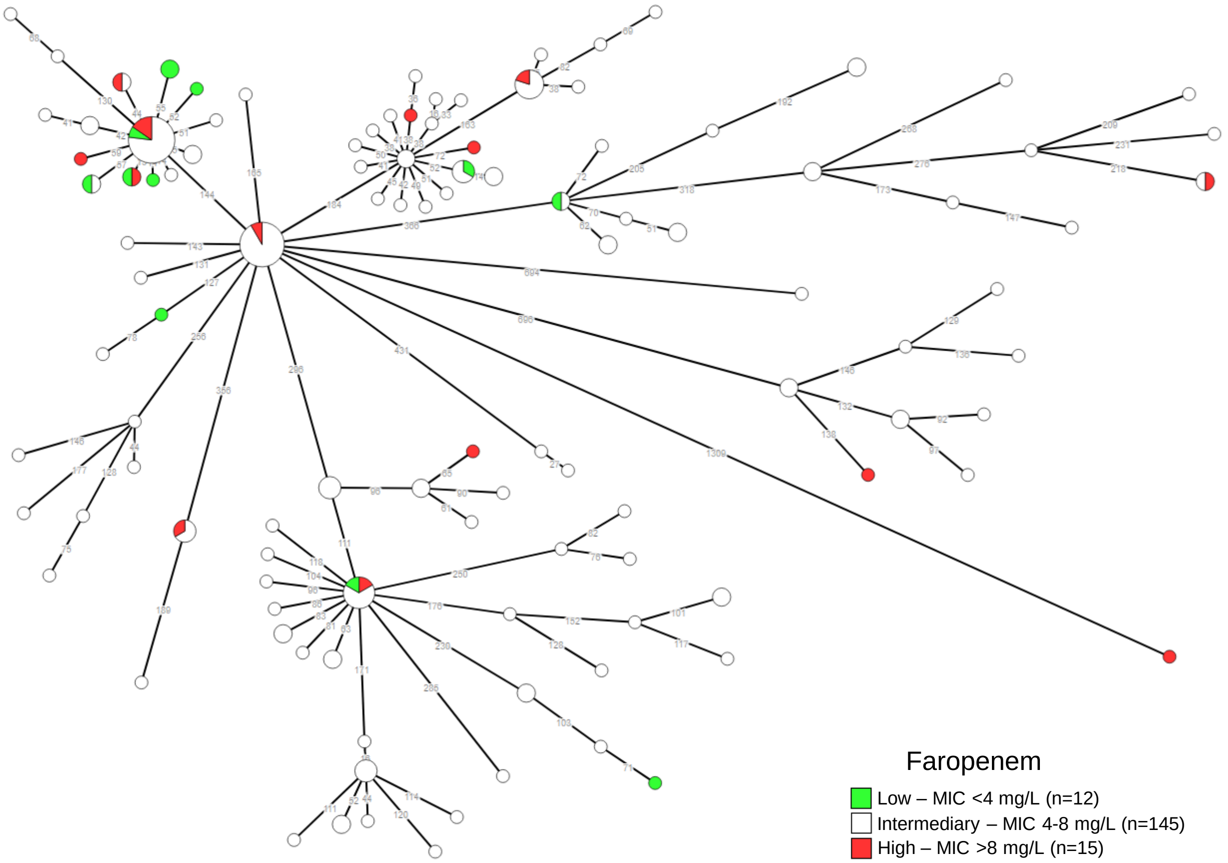

Note: No strains were selected for the second Mann-Whitney U test for faropenem since the first Mann-Whitney U test for this treatment did not yield any significant p-values (<0.05).

l

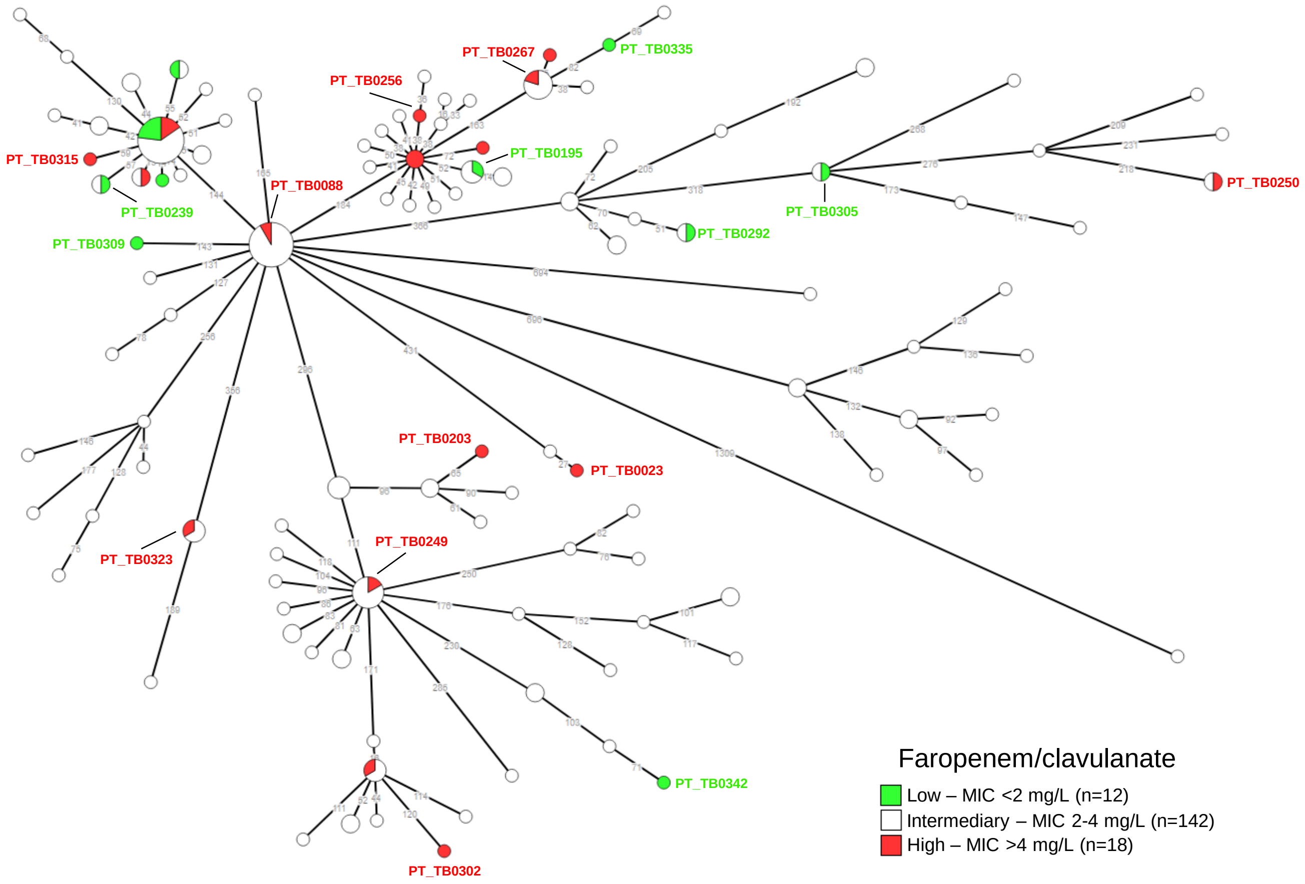

m

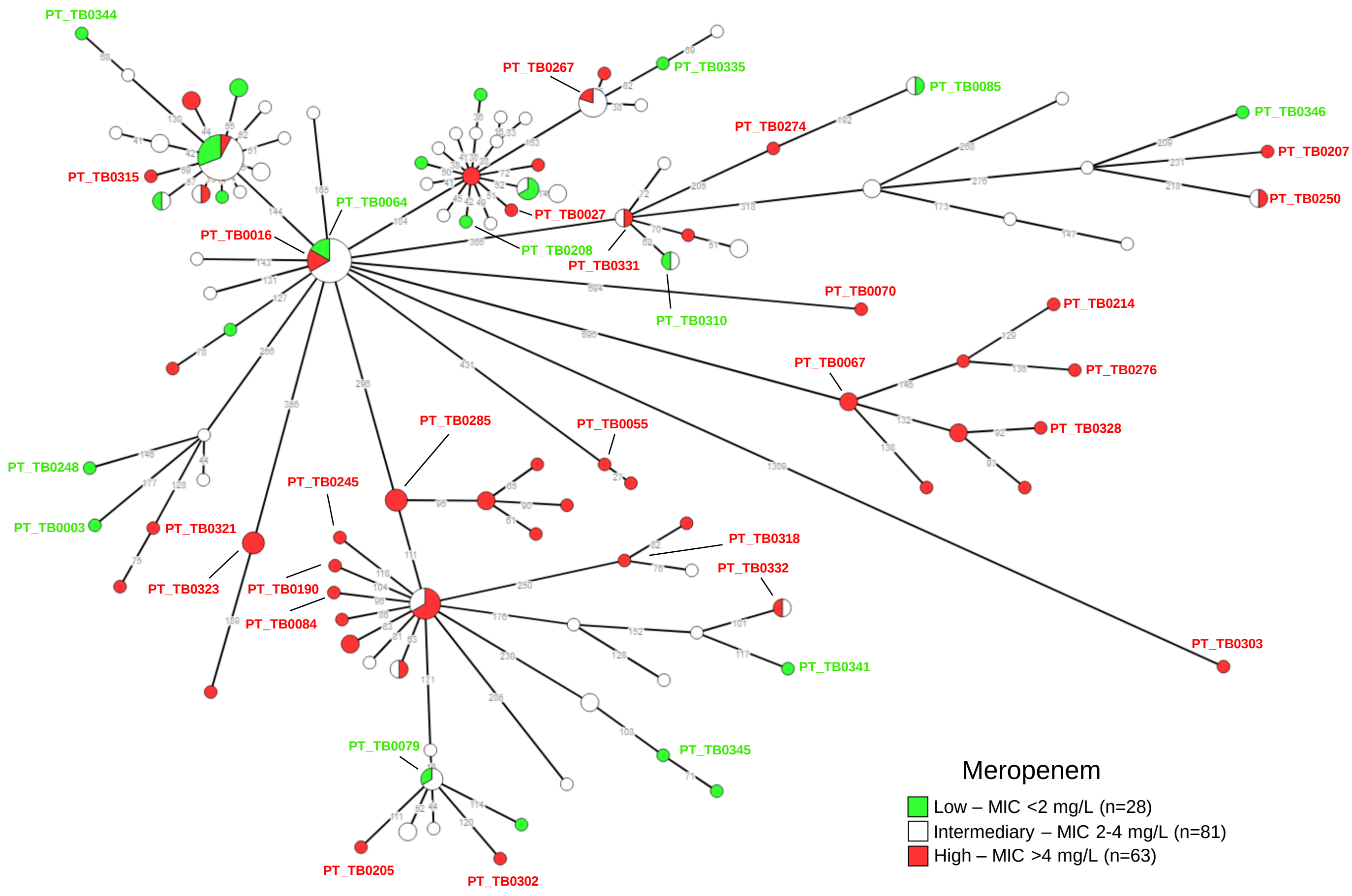
PT_TB0344
PT_TB0315
PT_TB0064
PT_TB0016
PT_TB0267
PT_TB0335
PT_TB0027
PT_TB0208
PT_TB0085
PT_TB0274
PT_TB0331
PT_TB0310
PT_TB0346
PT_TB0207
PT_TB0250
PT_TB0070
PT_TB0214
PT_TB0067
PT_TB0276
PT_TB0328
PT_TB0248
PT_TB0003
PT_TB0321
PT_TB0323
PT_TB0285
PT_TB0055
PT_TB0245
PT_TB0190
PT_TB0084
PT_TB0318
PT_TB0332
PT_TB0341
PT_TB0303
PT_TB0079
PT_TB0345
PT_TB0205
PT_TB0302
Meropenem
Low – MIC <2 mg/L (n=28)
Intermediary – MIC 2-4 mg/L (n=81)
High – MIC >4 mg/L (n=63)

n

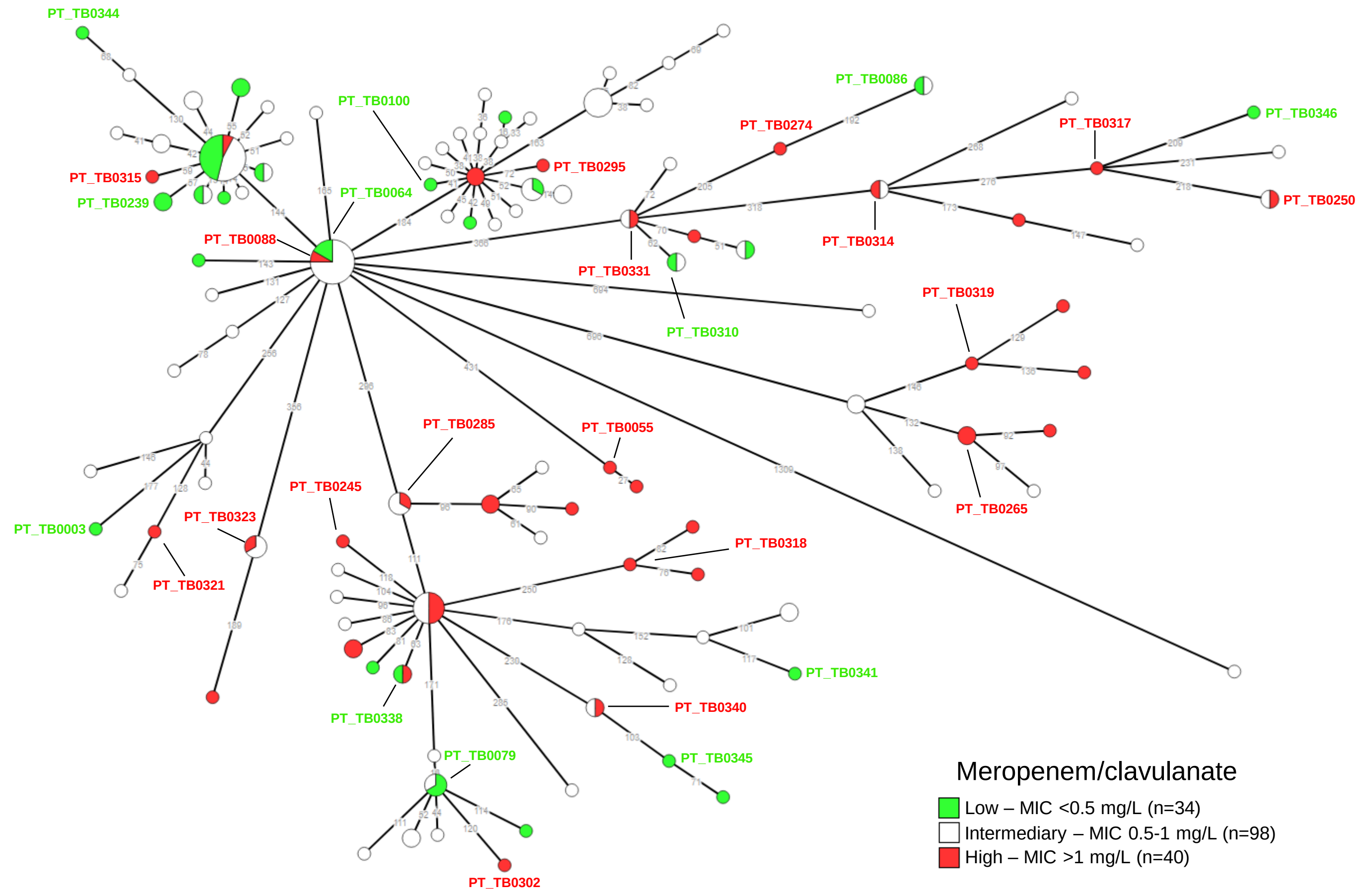
PT_TB0344
PT_TB0100
PT_TB0315
PT_TB0239
PT_TB0064
PT_TB0088
PT_TB0295
PT_TB0086
PT_TB0274
PT_TB0317
PT_TB0346
PT_TB0250
PT_TB0331
PT_TB0314
PT_TB0310
PT_TB0319
PT_TB0285
PT_TB0055
PT_TB0265
PT_TB0003
PT_TB0323
PT_TB0245
PT_TB0321
PT_TB0318
PT_TB0341
PT_TB0338
PT_TB0340
PT_TB0079
PT_TB0345
PT_TB0302
Meropenem/clavulanate
Low – MIC <0.5 mg/L (n=34)
Intermediary – MIC 0.5-1 mg/L (n=98)
High – MIC >1 mg/L (n=40)

Strain ID	SRA/ENA Accession #	Classification	Drug-resistance Profile	Antimycobacterial resistance											TB Profiler *in silico* prediction		
				INH	RIF	EMB	PZA	STR	LVX	MXF	AMK	KAN	ETI	Total	Lineage	Spoligotype	RD
PT_TB0064	ERR2864253	Resistant	Pre-XDR	•	•	•	•	•	•	•	•	•	•	10	lineage4.3.4.2	LAM1;LAM4;LAM11	RD174
PT_TB0061	ERR2864271	Resistant	Pre-XDR	•	•	•	•	•	•	•	•	•	•	10	lineage4.3.4.2	LAM1;LAM4;LAM11	RD174
PT_TB0013	ERR2864225	Resistant	Pre-XDR	•	•	•	•	•	•	•	•	•	•	10	lineage4.3.4.2	LAM1;LAM4;LAM11	RD174
PT_TB0015	ERR2864240	Resistant	Pre-XDR	•	•	•	•	•	•	•	•	•	•	10	lineage4.3.4.2	LAM1;LAM4;LAM11	RD174
PT_TB0088	ERR2864284	Resistant	Pre-XDR	•	•	•	•	•	•	•	•	•	•	10	lineage4.3.4.2	LAM1;LAM4;LAM11	RD174
PT_TB0217	ERR5530525	Resistant	Pre-XDR	•	•	•	•	•	•	•	•	•	•	10	lineage4.3.4.2	LAM1;LAM4;LAM11	RD174
PT_TB0087	ERR2864287	Resistant	Pre-XDR	•	•	•	•	•	•	•		•	•	9	lineage4.3.4.2	LAM1;LAM4;LAM11	RD174
PT_TB0099	ERR2984797	Resistant	Pre-XDR	•	•	•	•	•	•	•		•	•	9	lineage4.3.4.2	LAM1;LAM4;LAM11	RD174
PT_TB0014	ERR2864234	Resistant	Pre-XDR	•	•	•	•	•	•	•		•	•	9	lineage4.3.4.2	LAM1;LAM4;LAM11	RD174
PT_TB0059	ERR2864283	Resistant	Pre-XDR	•	•	•	•	•	•	•	•		•	9	lineage4.3.4.2	LAM1;LAM4;LAM11	RD174
PT_TB0058	ERR2864293	Resistant	Pre-XDR	•	•	•	•	•	•	•		•	•	9	lineage4.3.4.2	LAM1;LAM4;LAM11	RD174
PT_TB0224	ERR6326071	Resistant	Pre-XDR	•	•	•	•	•	•	•		•	•	9	lineage4.3.4.2	LAM1;LAM4;LAM11	RD174
PT_TB0072	ERR2864277	Resistant	Pre-XDR	•	•	•		•	•	•		•	•	8	lineage4.3.4.2	LAM1;LAM4;LAM11	RD174
PT_TB0017	ERR2864250	Resistant	MDR	•	•	•	•	•			•	•	•	8	lineage4.3.4.2	LAM1;LAM4;LAM11	RD174
PT_TB0214	ERR4781456	Resistant	Pre-XDR	•	•	•	•	•	•	•				7	lineage2.2.1.1	Beijing-RD150	RD105;RD207;RD181;RD150
PT_TB0003	ERR2864247	Resistant	MDR	•	•	•	•	•				•	•	7	lineage4.3.3	LAM;T	RD115
PT_TB0055	ERR2864220	Resistant	MDR	•	•	•	•	•				•	•	7	lineage4.2.1	H3;H4	None
PT_TB0023	ERR2864242	Resistant	MDR	•	•	•	•	•			•	•		7	lineage4.2.1	H3;H4	None
PT_TB0057	ERR2864286	Resistant	MDR	•	•	•	•	•				•		6	lineage2.2.1	Beijing-RD181	RD105;RD207;RD181
PT_TB0060	ERR2864227	Resistant	MDR	•	•	•	•	•					•	6	lineage4.3.2	LAM3	None
PT_TB0002	ERR2864288	Resistant	MDR	•	•	•	•	•					•	6	lineage4.3.4.2	LAM1;LAM4;LAM11	RD174
PT_TB0012	ERR2864218	Resistant	MDR	•	•	•	•	•					•	6	lineage4.3.4.2	LAM1;LAM4;LAM11	RD174
PT_TB0016	ERR2864264	Resistant	MDR	•	•	•	•	•					•	6	lineage4.3.4.2	LAM1;LAM4;LAM11	RD174
PT_TB0089	ERR2864281	Resistant	MDR	•	•		•	•					•	5	lineage4.3.4.2	LAM1;LAM4;LAM11	RD174
PT_TB0020	ERR2864292	Resistant	MDR	•	•	•		•					•	5	lineage4.3.4.2	LAM1;LAM4;LAM11	RD174
PT_TB0068	ERR2864245	Resistant	MDR	•	•	•		•					•	5	lineage4.3.4.2	LAM1;LAM4;LAM11	RD174
PT_TB0067	ERR2864255	Resistant	MDR	•	•	•		•					•	5	lineage2.2.1	Beijing-RD181	RD105;RD207;RD181
PT_TB0065	ERR2864268	Resistant	MDR	•	•		•	•					•	5	lineage4.3.4.2	LAM1;LAM4;LAM11	RD174
PT_TB0073	ERR2864273	Resistant	MDR	•	•		•	•					•	5	lineage4.3.4.2	LAM1;LAM4;LAM11	RD174
PT_TB0026	ERR2864257	Resistant	MDR	•	•	•		•					•	5	lineage4.3.4.2	LAM1;LAM4;LAM11	RD174
PT_TB0063	ERR2864275	Resistant	MDR	•	•	•	•	•						5	lineage4.1.2.1	T1;H1	RD182
PT_TB0062	ERR2864229	Resistant	MDR	•	•		•	•					•	5	lineage4.3.4.2	LAM1;LAM4;LAM11	RD174
PT_TB0008	ERR2864263	Resistant	MDR	•	•		•	•					•	5	lineage4.3.4.2	LAM1;LAM4;LAM11	RD174
PT_TB0022	ERR2864256	Resistant	MDR	•	•	•		•					•	5	lineage4.3.4.2	LAM1;LAM4;LAM11	RD174
PT_TB0025	ERR2864270	Resistant	MDR	•	•	•	•						•	5	lineage4.1	T;X;H	None
PT_TB0069	ERR2864228	Resistant	MDR	•	•	•		•					•	5	lineage2.2.1	Beijing-RD181	RD105;RD207;RD181
PT_TB0348	ERR7764396	Resistant	MDR	•	•	•	•	•						5	lineage4.3.4.2	LAM1;LAM4;LAM11	RD174
PT_TB0341	ERR7764348	Resistant	MDR	•	•	•	•	•						5	lineage4.3.2	LAM3	None
PT_TB0086	ERR2864274	Resistant	MDR	•	•	•		•						4	lineage4.7	T1;T5	None
PT_TB0081	ERR2864251	Resistant	MDR	•	•			•					•	4	lineage4.3.4.2	LAM1;LAM4;LAM11	RD174
PT_TB0085	ERR2864282	Resistant	MDR	•	•	•		•						4	lineage4.7	T1;T5	None
PT_TB0084	ERR2864266	Resistant	MDR	•	•	•		•						4	lineage4.1.2.1	T1;H1	RD182
PT_TB0100	ERR2984783	Resistant	MDR	•	•	•							•	4	lineage4.3.4.1	LAM1;LAM2	RD174
PT_TB0029	ERR2864231	Resistant	MDR	•	•	•							•	4	lineage2.2.1	Beijing-RD181	RD105;RD207;RD181
PT_TB0070	ERR2864232	Resistant	MDR	•	•			•					•	4	lineage3	CAS	RD750
PT_TB0027	ERR2864254	Resistant	MDR	•	•	•	•							4	lineage4.3.4.1	LAM1;LAM2	RD174
PT_TB0315	ERR7764342	Resistant	MDR	•	•	•		•						4	lineage4.3.4.2	LAM1;LAM4;LAM11	RD174
PT_TB0350	ERR7764370	Resistant	MDR	•	•	•		•						4	lineage4.3.4.2	LAM1;LAM4;LAM11	RD174
PT_TB0342	ERR7764327	Resistant	MDR	•	•	•	•							4	lineage4.1.1.1	X2	RD183
PT_TB0079	ERR2864222	Resistant	MDR	•	•			•						3	lineage4.1.1.3	X1;X3	RD193
PT_TB0071	ERR2864258	Resistant	MDR	•	•			•						3	lineage4.1.1.3	X1;X3	RD193
PT_TB0056	ERR2864243	Resistant	MDR	•	•			•						3	lineage4.1.1.3	X1;X3	RD193
PT_TB0190	ERR4352122	Resistant	MDR	•	•								•	3	lineage4.1.2.1	T1;H1	RD182
PT_TB0203	ERR4676897	Resistant	MDR	•	•			•						3	lineage4.1.2.1	T1;H1	RD182
PT_TB0209	ERR4676886	Resistant	Poly-drug resistant	•				•					•	3	lineage4.3.4.2	LAM1;LAM4;LAM11	RD174
PT_TB0074	ERR2864285	Resistant	MDR	•	•									2	lineage4.1.1.3	X1;X3	RD193
PT_TB0028	ERR2864295	Resistant	MDR	•	•									2	lineage4.3.4.1	LAM1;LAM2	RD174
PT_TB0205	ERR4676893	Resistant	MDR	•	•									2	lineage4.1.1.3	X1;X3	RD193
PT_TB0343	ERR7764393	Resistant	MDR	•	•									2	lineage4.3.4.1	LAM1;LAM2	RD174
PT_TB0258	ERR7764350	Resistant	Poly-drug resistant	•				•						2	lineage4.3.4.2	LAM1;LAM4;LAM11	RD174
PT_TB0259	ERR7764328	Resistant	Poly-drug resistant	•				•						2	lineage4.3.4.1	LAM1;LAM2	RD174
PT_TB0286	ERR7764337	Resistant	Poly-drug resistant	•				•						2	lineage4.3.4.2	LAM1;LAM4;LAM11	RD174
PT_TB0207	ERR4676882	Resistant	Poly-drug resistant	•									•	2	lineage4.8	T1;T2;T3;T5	RD219
PT_TB0345	ERR7764395	Resistant	Mono-drug resistant					•						1	lineage4.1.1.1	X2	RD183
PT_TB0344	ERR7764329	Resistant	Mono-drug resistant	•										1	lineage4.3.4.2	LAM1;LAM4;LAM11	RD174
PT_TB0208	ERR4676894	Resistant	Mono-drug resistant	•										1	lineage4.3.4.1	LAM1;LAM2	RD174
PT_TB0320	ERR7764363	Resistant	Mono-drug resistant					•						1	lineage4.3.4.2	LAM1;LAM4;LAM11	RD174
PT_TB0246	ERR7764357	Resistant	Mono-drug resistant					•						1	lineage4.3.4.2	LAM1;LAM4;LAM11	RD174
PT_TB0280	ERR7764394	Resistant	Mono-drug resistant					•						1	lineage4.8	T1;T2;T3;T5	RD219
PT_TB0295	ERR7764409	Resistant	Mono-drug resistant					•						1	lineage4.3.4.1	LAM1;LAM2	RD174
PT_TB0302	ERR7764424	Resistant	Mono-drug resistant					•						1	lineage4.1.1.3	X1;X3	RD193
PT_TB0305	ERR7764375	Resistant	Mono-drug resistant					•						1	lineage4.8	T1;T2;T3;T5	RD219
PT_TB0346	ERR7764414	Susceptible	Susceptible											0	lineage4.8	T1;T2;T3;T5	RD219
PT_TB0329	ERR7764402	Susceptible	Susceptible											0	lineage4.3.4.2	LAM1;LAM4;LAM11	RD174
PT_TB0331	ERR7764376	Susceptible	Susceptible											0	lineage4.7	T1;T5	None
PT_TB0338	ERR7764429	Susceptible	Susceptible											0	lineage4.1.2.1	T1;H1	RD182
PT_TB0340	ERR7764432	Susceptible	Susceptible											0	lineage4.1.1.1	X2	RD183
PT_TB0337	ERR7764352	Susceptible	Susceptible											0	lineage4.1.2.1	T1;H1	RD182
PT_TB0339	ERR7764410	Susceptible	Susceptible											0	lineage4.1.2.1	T1;H1	RD182
PT_TB0307	ERR7764358	Susceptible	Susceptible											0	lineage4.1.2	T;H	None
PT_TB0310	ERR7764365	Susceptible	Susceptible											0	lineage4.7	T1;T5	None
PT_TB0311	ERR7764372	Susceptible	Susceptible											0	lineage4.8	T1;T2;T3;T5	RD219
PT_TB0312	ERR7764436	Susceptible	Susceptible											0	lineage4.3.4.2	LAM1;LAM4;LAM11	RD174
PT_TB0314	ERR7764364	Susceptible	Susceptible											0	lineage4.8	T1;T2;T3;T5	RD219
PT_TB0309	ERR7764381	Susceptible	Susceptible											0	lineage4.3.4.2	LAM1;LAM4;LAM11	RD174
PT_TB0308	ERR7764332	Susceptible	Susceptible											0	lineage4.3.4.1	LAM1;LAM2	RD174

Table S1. Information of the Sequence Read Archive (SRA)/European Nucleotide Archive (ENA) accession numbers, antimycobacterial drug susceptibility testing and *in silico* TB-profiler predictions of lineages, spoligotypes and regions of difference (RD) of the *Mtb* clinical isolates included in this study. Total indicates the number of antimycobacterial drugs a given strain was resistant to. The filled circle symbol (●) indicates resistance to the respective antibiotic. AMK, amikacin; EMB, ethambutol; ETI, ethionamide; INH, isoniazid; KAN, kanamycin; LVX, levofloxacin; MXF, moxifloxacin; PZA, pyrazinamide; RIF, rifampicin; STR, streptomycin.

Strain ID	SRA/ENA Accession #	Classification	Drug-resistance Profile	Antimycobacterial resistance											TB Profiler *in silico* prediction		
				INH	RIF	EMB	PZA	STR	LVX	MXF	AMK	KAN	ETI	Total	Lineage	Spoligotype	RD
PT_TB0336	ERR7764331	Susceptible	Susceptible											0	lineage4.3.4.2	LAM1;LAM4;LAM11	RD174
PT_TB0335	ERR7764371	Susceptible	Susceptible											0	lineage4.3.4.1	LAM1;LAM2	RD174
PT_TB0334	ERR7764440	Susceptible	Susceptible											0	lineage4.1.2.1	T1;H1	RD182
PT_TB0333	ERR7764359	Susceptible	Susceptible											0	lineage4.1.2.1	T1;H1	RD182
PT_TB0332	ERR7764330	Susceptible	Susceptible											0	lineage4.3.2	LAM3	None
PT_TB0330	ERR7764407	Susceptible	Susceptible											0	lineage4.1.1.1	X2	RD183
PT_TB0328	ERR7764343	Susceptible	Susceptible											0	lineage2.2.1	Beijing-RD181	RD105;RD207;RD181
PT_TB0327	ERR7764433	Susceptible	Susceptible											0	lineage4.3.4.1	LAM1;LAM2	RD174
PT_TB0326	ERR7764422	Susceptible	Susceptible											0	lineage4.3.2	LAM3	None
PT_TB0325	ERR7764435	Susceptible	Susceptible											0	lineage4.4.1.1	S;Orphans	None
PT_TB0324	ERR7764360	Susceptible	Susceptible											0	lineage4.4.1.1	S;Orphans	None
PT_TB0323	ERR7764389	Susceptible	Susceptible											0	lineage4.4.1.1	S;Orphans	None
PT_TB0322	ERR7764344	Susceptible	Susceptible											0	lineage4.3.4.1	LAM1;LAM2	RD174
PT_TB0321	ERR7764411	Susceptible	Susceptible											0	lineage4.3.3	LAM;T	RD115
PT_TB0319	ERR7764417	Susceptible	Susceptible											0	lineage2.2.1.1	Beijing-RD150	RD105;RD207;RD181;RD150
PT_TB0318	ERR7764418	Susceptible	Susceptible											0	lineage4.1.2	T;H	None
PT_TB0317	ERR7764338	Susceptible	Susceptible											0	lineage4.8	T1;T2;T3;T5	RD219
PT_TB0313	ERR7764325	Susceptible	Susceptible											0	lineage4.8	T1;T2;T3;T5	RD219
PT_TB0316	ERR7764405	Susceptible	Susceptible											0	lineage4.3.4.2	LAM1;LAM4;LAM11	RD174
PT_TB0244	ERR7764366	Susceptible	Susceptible											0	lineage4.7	T1;T5	None
PT_TB0245	ERR7764426	Susceptible	Susceptible											0	lineage4.1.2.1	T1;H1	RD182
PT_TB0247	ERR7764403	Susceptible	Susceptible											0	lineage4.3.4.1	LAM1;LAM2	RD174
PT_TB0248	ERR7764412	Susceptible	Susceptible											0	lineage4.3.3	LAM;T	RD115
PT_TB0249	ERR7764377	Susceptible	Susceptible											0	lineage4.1.2.1	T1;H1	RD182
PT_TB0250	ERR7764333	Susceptible	Susceptible											0	lineage4.8	T1;T2;T3;T5	RD219
PT_TB0251	ERR7764367	Susceptible	Susceptible											0	lineage4.1.2.1	T1;H1	RD182
PT_TB0252	ERR7764437	Susceptible	Susceptible											0	lineage4.3.4.2	LAM1;LAM4;LAM11	RD174
PT_TB0253	ERR7764374	Susceptible	Susceptible											0	lineage4.3.4.1	LAM1;LAM2	RD174
PT_TB0254	ERR7764336	Susceptible	Susceptible											0	lineage4.1.2.1	T1;H1	RD182
PT_TB0255	ERR7764415	Susceptible	Susceptible											0	lineage4.1.2	T;H	None
PT_TB0256	ERR7764349	Susceptible	Susceptible											0	lineage4.3.4.1	LAM1;LAM2	RD174
PT_TB0257	ERR7764399	Susceptible	Susceptible											0	lineage4.3.4.1	LAM1;LAM2	RD174
PT_TB0260	ERR7764391	Susceptible	Susceptible											0	lineage4.7	T1;T5	None
PT_TB0261	ERR7764341	Susceptible	Susceptible											0	lineage4.3.2	LAM3	None
PT_TB0263	ERR7764351	Susceptible	Susceptible											0	lineage4.3.4.1	LAM1;LAM2	RD174
PT_TB0262	ERR7764404	Susceptible	Susceptible											0	lineage4.3.4.1	LAM1;LAM2	RD174
PT_TB0267	ERR7764392	Susceptible	Susceptible											0	lineage4.3.4.1	LAM1;LAM2	RD174
PT_TB0266	ERR7764387	Susceptible	Susceptible											0	lineage4.1.2.1	T1;H1	RD182
PT_TB0269	ERR7764384	Susceptible	Susceptible											0	lineage4.1.2.1	T1;H1	RD182
PT_TB0268	ERR7764368	Susceptible	Susceptible											0	lineage4.1.1.3	X1;X3	RD193
PT_TB0273	ERR7764431	Susceptible	Susceptible											0	lineage4.3.4.2	LAM1;LAM4;LAM11	RD174
PT_TB0272	ERR7764439	Susceptible	Susceptible											0	lineage4.1.2.1	T1;H1	RD182
PT_TB0279	ERR7764434	Susceptible	Susceptible											0	lineage4.3.4.1	LAM1;LAM2	RD174
PT_TB0276	ERR7764390	Susceptible	Susceptible											0	lineage2.2.1	Beijing-RD181	RD105;RD207;RD181
PT_TB0281	ERR7764345	Susceptible	Susceptible											0	lineage4.3.4.1	LAM1;LAM2	RD174
PT_TB0284	ERR7764324	Susceptible	Susceptible											0	lineage4.3.4.2	LAM1;LAM4;LAM11	RD174
PT_TB0264	ERR7764323	Susceptible	Susceptible											0	lineage4.3.4.1	LAM1;LAM2	RD174
PT_TB0271	ERR7764401	Susceptible	Susceptible											0	lineage4.1.2.1	T1;H1	RD182
PT_TB0270	ERR7764385	Susceptible	Susceptible											0	lineage4.1.1.3	X1;X3	RD193
PT_TB0265	ERR7764438	Susceptible	Susceptible											0	lineage2.2.1	Beijing-RD181	RD105;RD207;RD181
PT_TB0274	ERR7764397	Susceptible	Susceptible											0	lineage4.7	T1;T5	None
PT_TB0275	ERR7764361	Susceptible	Susceptible											0	lineage4.3.4.2	LAM1;LAM4;LAM11	RD174
PT_TB0278	ERR7764420	Susceptible	Susceptible											0	lineage4.3.4.1	LAM1;LAM2	RD174
PT_TB0277	ERR7764355	Susceptible	Susceptible											0	lineage4.3.2	LAM3	None
PT_TB0283	ERR7764326	Susceptible	Susceptible											0	lineage4.3.4.2	LAM1;LAM4;LAM11	RD174
PT_TB0285	ERR7764388	Susceptible	Susceptible											0	lineage4.1.2.1	T1;H1	RD182
PT_TB0289	ERR7764419	Susceptible	Susceptible											0	lineage4.3.4.2	LAM1;LAM4;LAM11	RD174
PT_TB0288	ERR7764362	Susceptible	Susceptible											0	lineage2.2.1	Beijing-RD181	RD105;RD207;RD181
PT_TB0292	ERR7764356	Susceptible	Susceptible											0	lineage4.7	T1;T5	None
PT_TB0291	ERR7764382	Susceptible	Susceptible											0	lineage4.3.4.1	LAM1;LAM2	RD174
PT_TB0296	ERR7764427	Susceptible	Susceptible											0	lineage4.3.4.1	LAM1;LAM2	RD174
PT_TB0299	ERR7764386	Susceptible	Susceptible											0	lineage4.3.3	LAM;T	RD115
PT_TB0306	ERR7764380	Susceptible	Susceptible											0	lineage4.1.1.3	X1;X3	RD193
PT_TB0297	ERR7764340	Susceptible	Susceptible											0	lineage4.7	T1;T5	None
PT_TB0293	ERR7764423	Susceptible	Susceptible											0	lineage4.7	T1;T5	None
PT_TB0294	ERR7764347	Susceptible	Susceptible											0	lineage4.1.2.1	T1;H1	RD182
PT_TB0287	ERR7764421	Susceptible	Susceptible											0	lineage4.1.2.1	T1;H1	RD182
PT_TB0282	ERR7764379	Susceptible	Susceptible											0	lineage4.3.3	LAM;T	RD115
PT_TB0300	ERR7764416	Susceptible	Susceptible											0	lineage4.1.2.1	T1;H1	RD182
PT_TB0298	ERR7764430	Susceptible	Susceptible											0	lineage4.1.2.1	T1;H1	RD182
PT_TB0301	ERR7764398	Susceptible	Susceptible											0	lineage4.1.2.1	T1;H1	RD182
PT_TB0304	ERR7764413	Susceptible	Susceptible											0	lineage4.7	T1;T5	None
PT_TB0303	ERR7764425	Susceptible	Susceptible											0	lineage6	AFRI 1	RD702
PT_TB0290	ERR7764354	Susceptible	Susceptible											0	lineage4.4.1.1	S;Orphans	None
PT_TB0240	ERR7764373	Susceptible	Susceptible											0	lineage4.3.4.2	LAM1;LAM4;LAM11	RD174
PT_TB0239	ERR7764400	Susceptible	Susceptible											0	lineage4.3.4.2	LAM1;LAM4;LAM11	RD174
PT_TB0241	ERR7764441	Susceptible	Susceptible											0	lineage4.3.4.2	LAM1;LAM4;LAM11	RD174
PT_TB0243	ERR7764408	Susceptible	Susceptible											0	lineage4.3.3	LAM;T	RD115
PT_TB0242	ERR7764378	Susceptible	Susceptible											0	lineage4.8	T1;T2;T3;T5	RD219
PT_TB0236	ERR7764369	Susceptible	Susceptible											0	lineage4.3.4.1	LAM1;LAM2	RD174
PT_TB0238	ERR7764339	Susceptible	Susceptible											0	lineage4.3.4.1	LAM1;LAM2	RD174
PT_TB0237	ERR7764334	Susceptible	Susceptible											0	lineage4.1.1.3	X1;X3	RD193
PT_TB0347	ERR7764353	Susceptible	Susceptible											0	lineage4.3.4.1	LAM1;LAM2	RD174
PT_TB0195	ERR4676884	Susceptible	Susceptible											0	lineage4.3.4.1	LAM1;LAM2	RD174
PT_TB0349	ERR7764383	Susceptible	Susceptible											0	lineage4.3.4.1	LAM1;LAM2	RD174
PT_TB0194	ERR4676901	Susceptible	Susceptible											0	lineage4.3.4.1	LAM1;LAM2	RD174

Table S1. (Continued) Information of the Sequence Read Archive (SRA)/European Nucleotide Archive (ENA) accession numbers, antimycobacterial drug susceptibility testing and *in silico* TB-profiler predictions of lineages, spoligotypes and regions of difference (RD) of the *Mtb* clinical isolates included in this study. Total indicates the number of antimycobacterial drugs a given strain was resistant to. The filled circle symbol (●) indicates resistance to the respective antibiotic. AMK, amikacin; EMB, ethambutol; ETI, ethionamide; INH, isoniazid; KAN, kanamycin; LVX, levofloxacin; MXF, moxifloxacin; PZA, pyrazinamide; RIF, rifampicin; STR, streptomycin.

Sublineage	Isolates (%)	AMX	AMX/CLA	MEM	MEM/CLA
4.3.4.2	50 (29.0)	**3.85E-08**	**5.68E-06**	**5.75E-04**	**3.89E-04**
4.3.4.1	30 (17.4)	7.76E-02	2.54E-01	**7.86E-03**	1.85E-01
4.1.2.1	23 (13.3)	**9.63E-06**	**4.40E-05**	**3.69E-06**	**7.77E-04**
4.7	11 (6.4)	9.36E-01	5.96E-01	3.14E-01	3.68E-01
2.2.1 & 2.2.1.1	10 (5.8)	**3.69E-04**	**7.31E-05**	**2.95E-05**	**2.95E-03**
4.8	10 (5.8)	1.86E-01	6.49E-01	6.79E-01	4.52E-01
4.1.1.3	10 (5.8)	9.81E-01	3.37E-01	3.85E-01	6.66E-01

Table S2. p-values obtained by the statistical analysis by Mann-Whitney U test of the differences in MIC distributions between specific subsets of strains from sublineages with 10 or more members and all other strains. This analysis was performed for amoxicillin (AMX), amoxicillin/clavulanate (AMX/CLA), meropenem (MEM) and meropenem/clavulanate (MEM/CLA). Strains from sublineages 2.2.1 and 2.2.1.1 were analysed together as Beijing strains. p-values below 0.05 (in bold) were considered significant.

Function	Locus Tag	Gene name	Genomic locus	Mutation	Effect in product	Number of isolates (%)	Sublineage	Drug-Resistance	AMX	AMX/CLA	CTX	CTX/CLA	FAR	FAR/CLA	BIA	BIA/CLA	DOR	DOR/CLA	MEM	MEM/CLA	ETP	ETP/CLA	Provean Score[a]
									48.3[b]	1.8[b]	10.7[b]	3.2[b]	5.5[b]	3.3[b]	3.9[b]	0.7[b]	5.3[b]	0.8[b]	4.0[b]	0.8[b]	33.3[b]	4.2[b]	
Beta-lactamase activity																							
	Rv1845c	*blaR*	2095691	C>T	G160S	1 (0.58)	4.1.2.1 (1)	MDR (1)	32.0	1.0	4.0	4.0	8.0	4.0	16.0	1.0	16.0	2.0	8.0	1.0	64.0	8.0	-5.746
	Rv2068c	*blaC*	2326239	C>T	G191R	1 (0.58)	4.8 (1)	Susceptible (1)	64.0	2.0	8.0	2.0	4.0	4.0	4.0	1.0	4.0	1.0	4.0	2.0	16.0	4.0	-6.184
			2326664	G>C	A49G	4 (2.33)	4.1.1.1 (4)	Susceptible (2)<br>Mono-resistant (1)<br>MDR (1)	22.6	1.0	2.8	1.0	4.0	2.4	1.7	0.6	2.4	0.6	1.7	0.6	13.5	2.0	-2.823
Function	Locus Tag	Gene name	Genomic locus	Mutation	Effect in product	Number of isolates (%)	Sublineage	Drug-Resistance	AMX	AMX/CLA	CTX	CTX/CLA	FAR	FAR/CLA	BIA	BIA/CLA	DOR	DOR/CLA	MEM	MEM/CLA	ETP	ETP/CLA	Provean Score[a]
									48.3[b]	1.8[b]	10.7[b]	3.2[b]	5.5[b]	3.3[b]	3.9[b]	0.7[b]	5.3[b]	0.8[b]	4.0[b]	0.8[b]	33.3[b]	4.2[b]	
PG synthesis																							
	Rv0482	*murB*	570657	C>T	T40I	1 (0.58)	2.2.1 (1)	MDR (1)	>64.0.0	>64.0	>64.0	>64.0	16.0	4.0	16.0	0.5	16.0	1.0	8.0	1.0	>64.0	8.0	-3.105
			570843	C>G	A102G	1 (0.58)	4.8 (1)	Mono-resistant (1)	64.0	1.0	8.0	2.0	4.0	2.0	4.0	0.5	4.0	0.5	2.0	0.5	32.0	4.0	-3.868
	Rv1018c	*glmU*	1137790	G>C	H91D	1 (0.58)	4.8(1)	Susceptible (1)	64.0	2.0	8.0	2.0	4.0	4.0	4.0	1.0	4.0	1.0	4.0	2.0	16.0	4.0	-3.855
	Rv1315	*murA*	1470495	C>T	R59C	1 (0.58)	4.4.1.1 (1)	Susceptible (1)	>64.0	2.0	16.0	4.0	4.0	4.0	16.0	1.0	32.0	1.0	32.0	1.0	64.0	8.0	-4.849
			1470649	G>C	R110T	2 (1.16)	4.2.1 (2)	MDR (2)	>64.0	16.0	11.3	5.7	5.7	5.7	16.0	2.8	22.6	2.8	45.3	4.0	32.0	16.0	-2.444
	Rv2152c	*murC*	2410831	T>C	H431R	4 (2.33)	4.8 (4)	Susceptible (3)<br>Mono-resistant (1)	>64.0	1.0	9.5	2.4	4.8	2.8	4.8	0.6	4.8	0.7	2.8	1.0	32.0	2.8	1.997
			2411537	G>C	L196V	1 (0.58)	2.2.1.1 (1)	Susceptible (1)	>64.0	64.0	16.0	8.0	8.0	4.0	32.0	4.0	64.0	4.0	64.0	4.0	>64.0	16.0	-2.992
			2411642	G>A	H161Y	1 (0.58)	4.3.4.2 (1)	Susceptible (1)	>64.0	2.0	32.0	8.0	8.0	4.0	16.0	1.0	8.0	1.0	8.0	1.0	64.0	8.0	-4.675
			2412086	C>T	V13M	1 (0.58)	4.3.4.1 (1)	Susceptible (1)	32.0	1.0	8.0	4.0	4.0	2.0	2.0	0.5	2.0	0.5	2.0	0.5	16.0	2.0	-2.774
	Rv2153c	*murG*	2412348	C>G	R335P	3 (1.74)	4.1.2.1 (3)	Susceptible (3)	>64.0	5.0	8.0	3.2	4.0	4.0	16.0	1.0	16.0	1.0	16.0	1.3	64.0	8.0	-3.321
	Rv2155c	*murD*	2415656	G>C	R247G	170 (98.84)	All except 4.1.1.3 (1) and 4.3.4.1 (1)	Susceptible (100)<br>Mono or Poly-resistant (13)<br>MDR (43)<br>Pre-XDR (14)	48.3	1.8	10.7	3.2	5.5	3.3	4.0	0.7	5.3	0.8	4.0	0.8	33.6	4.3	5.985
			2416156	G>A	T80I	41 (23.84)	4.1 (1)<br>4.1.1.1 (4)<br>4.1.1.3 (10)<br>4.1.2 (3)<br>4.1.2.1 (23)	Susceptible (28)<br>Mono-resistant (2)<br>MDR (11)	>64.0	2.8	10.5	3.4	5.1	3.5	5.6	1.0	8.0	1.0	5.9	1.0	44.9	4.9	-2.192
			2416167	G>T	F76L	4 (2.33)	4.4.1.1 (4)	Susceptible (4)	>64.0	5.7	26.9	11.3	6.7	4.8	22.6	1.7	22.6	1.7	16.0	2.0	>64.0	9.5	-4.473
	Rv2156c	*murX*	2417468	T>A	Q3L	1 (0.58)	4.1 (1)	MDR (1)	64.0	2.0	16.0	4.0	4.0	4.0	2.0	0.3	2.0	0.5	4.0	0.5	16.0	4.0	-4.438
	Rv2157c	*murF*	2418194	T>C	N271D	2 (1.16)	4.1.1.3 (2)	Susceptible (2)	45.3	1.0	2.8	1.0	4.0	4.0	2.8	0.7	8.0	0.7	2.8	0.7	64.0	5.7	-0.408
			2418394	G>C	P204R	2 (1.16)	4.2.1 (2)	MDR (2)	>64.0	16.0	11.3	5.7	5.7	5.7	16.0	2.8	22.6	2.8	45.3	4.0	32.0	16.0	-7.829
	Rv2158c	*murE*	2420535	C>A	G25V	50 (29.07)	4.3.4.2 (50)	Susceptible (14)<br>Mono or Poly-resistant (6)<br>MDR (17)<br>Pre-XDR (13)	25.6	0.9	6.2	1.7	5.2	3.1	2.6	0.5	3.5	0.6	2.5	0.5	18.9	2.8	-0.922
	Rv2981c	*ddl*	3336825	T>C	T365A	151 (87.79)	All except 4.7 (11) and 4.8 (10)	Susceptible (84)<br>Mono or Poly-resistant (11)<br>MDR (42)<br>Pre-XDR (14)	46.6	1.8	10.5	3.1	5.5	3.3	3.9	0.7	5.3	0.8	4.1	0.8	33.3	4.1	3.946
Function	Locus Tag	Gene name	Genomic locus	Mutation	Effect in product	Number of isolates (%)	Sublineage	Drug-Resistance	AMX	AMX/CLA	CTX	CTX/CLA	FAR	FAR/CLA	BIA	BIA/CLA	DOR	DOR/CLA	MEM	MEM/CLA	ETP	ETP/CLA	Provean Score[a]
									48.3[b]	1.8[b]	10.7[b]	3.2[b]	5.5[b]	3.3[b]	3.9[b]	0.7[b]	5.3[b]	0.8[b]	4.0[b]	0.8[b]	33.3[b]	4.2[b]	
PG assembly																							
	Rv0016c	*pbpA*	19655	G>C	Q194E	1 (0.58)	4.3.4.1 (1)	Susceptible (1)	32.0	2.0	>64.0	8.0	8.0	4.0	2.0	0.5	4.0	1.0	4.0	1.0	>64.0	4.0	-0.877
	Rv0050	*ponA1*	54239	C>G	R193G	6 (3.49)	4.3.4.1 (6)	Susceptible (6)	50.8	1.8	20.2	6.3	9.0	4.5	2.8	0.5	3.2	0.8	3.2	0.7	50.8	5.7	-2.144
			54757	G>T	Q365H	1 (0.58)	2.2.1 (1)	MDR (1)	>64.0	>64.0	>64.0	>64.0	16.0	4.0	16.0	0.5	16.0	1.0	8.0	1.0	>64.0	8.0	-2.134
			54842	G>T	A394S	1 (0.58)	3 (1)	MDR (1)	>64.0	4.0	16.0	2.0	8.0	4.0	16.0	2.0	32.0	2.0	16.0	1.0	>64.0	16.0	-2.965
			55549	GCCGC>TCCGCCGCCGCCGT	P631_E632insPPS	8 (4.65)	2.2.1 (6)<br>2.2.1.1 (1)<br>3 (1)	Susceptible (5)<br>MDR (3)	>64.0	8.0	19.0	4.8	6.2	2.8	17.4	1.7	24.7	1.8	19.0	1.7	>64.0	16.0	2.692
	Rv0116c	*ldtA*	140644	C>T	G127R	1 (0.58)	6 (1)	Susceptible (1)	>64.0	2.0	16.0	4.0	32.0	2.0	8.0	1.0	64.0	2.0	8.0	0.5	4.0	1.0	-6.408
	Rv0192	*ldtE*	223885	C>G	P108A	1 (0.58)	4.3.4.2 (1)	Pre-XDR (1)	64.0	2.0	32.0	4.0	8.0	4.0	4.0	0.5	8.0	1.0	4.0	1.0	64.0	2.0	-0.131
			223942	T>C	S127P	172 (100.00)	All	All	48.3	1.8	10.7	3.2	5.5	3.3	3.9	0.7	5.3	0.8	4.0	0.8	33.3	4.2	3.959
			224338	C>T	P259S	1 (0.58)	3 (1)	MDR (1)	>64.0	4.0	16.0	2.0	8.0	4.0	16.0	2.0	32.0	2.0	16.0	1.0	>64.0	16.0	-3.746
			224467	A>T	S302C	1 (0.58)	4.1.1.3 (1)	Susceptible (1)	64.0	0.5	4.0	1.0	4.0	2.0	2.0	0.5	4.0	0.5	2.0	1.0	64.0	4.0	-4.299
	Rv1433	*ldtD*	1612184	C>G	R251G	1 (0.58)	4.3.4.2 (1)	Mono-resistant (1)	16.0	0.3	2.0	1.0	8.0	2.0	4.0	0.5	2.0	1.0	0.5	0.3	32.0	4.0	-0.222
	Rv2163c	*pbpB*	2426439	C>T	A217T	3 (1.74)	4.1.2.1 (3)	Susceptible (3)	>64.0	5.0	8.0	3.2	4.0	4.0	16.0	1.0	16.0	1.0	16.0	1.3	64.0	8.0	-2.133
			2426554	G>C	D178E	1 (0.58)	4.3.4.2 (1)	MDR (1)	32.0	1.0	4.0	1.0	8.0	4.0	4.0	0.5	4.0	0.5	4.0	0.5	16.0	2.0	-1.168
	Rv2518c	*ldtB*	2835261	C>T	M25I	1 (0.58)	6 (1)	Susceptible (1)	>64.0	2.0	16.0	4.0	32.0	2.0	8.0	1.0	64.0	2.0	8.0	0.5	4.0	1.0	0.758
	Rv2911	*dacB2*	3218343	G>A	R2Q	6 (3.49)	4.3.3 (6)	Susceptible (5)<br>MDR (1)	50.8	1.8	25.4	6.3	6.3	3.6	3.2	0.7	4.0	0.6	3.2	0.6	28.5	4.0	-0.451
			3218672	A>G	T112A	1 (0.58)	4.1 (1)	MDR (1)	64.0	2.0	16.0	4.0	4.0	4.0	2.0	0.3	2.0	0.5	4.0	0.5	16.0	4.0	-4.083
			3219129	G>A	G264D	2 (1.16)	2.2.1 (2)	Susceptible (2)	>64.0	8.0	22.6	5.7	4.0	2.0	16.0	1.4	16.0	2.0	16.0	2.8	>64.0	16.0	-1.487
	Rv3627	-	4067044	G>A	A81V	1 (0.58)	6 (1)	Susceptible (1)	>64.0	2.0	16.0	4.0	32.0	2.0	8.0	1.0	64.0	2.0	8.0	0.5	4.0	1.0	-2.832
			4067152	G>A	A45V	1 (0.58)	6 (1)	Susceptible (1)	>64.0	2.0	16.0	4.0	32.0	2.0	8.0	1.0	64.0	2.0	8.0	0.5	4.0	1.0	-1.100
	Rv3682	*ponA2*	4122477	A>G	T188A	3 (1.74)	4.3.4.1 (3)	Susceptible (2)<br>MDR (1)	20.2	0.4	4.0	1.3	3.2	1.6	1.0	0.4	1.0	0.4	1.3	0.4	20.2	2.5	-3.521
			4122979	T>C	V355A	1 (0.58)	6 (1)	Susceptible (1)	>64.0	2.0	16.0	4.0	32.0	2.0	8.0	1.0	64.0	2.0	8.0	0.5	4.0	1.0	-3.398
			4124254	C>G	P780R	1 (0.58)	3 (1)	MDR (1)	>64.0	4.0	16.0	2.0	8.0	4.0	16.0	2.0	32.0	2.0	16.0	1.0	>64.0	16.0	-1.466
Function	Locus Tag	Gene name	Genomic locus	Mutation	Effect in product	Number of isolates (%)	Sublineage	Drug-Resistance	AMX	AMX/CLA	CTX	CTX/CLA	FAR	FAR/CLA	BIA	BIA/CLA	DOR	DOR/CLA	MEM	MEM/CLA	ETP	ETP/CLA	Provean Score[a]
									48.3[b]	1.8[b]	10.7[b]	3.2[b]	5.5[b]	3.3[b]	3.9[b]	0.7[b]	5.3[b]	0.8[b]	4.0[b]	0.8[b]	33.3[b]	4.2[b]	
PG hydrolysis																							
	Rv0320	-	388300	A>G	H138R	1 (0.58)	4.7 (1)	Susceptible (1)	32.0	1.0	8.0	2.0	4.0	2.0	1.0	0.3	2.0	0.5	2.0	0.5	16.0	4.0	-8.000
	Rv0867c	*rpfA*	965247	C>T	A97T	1 (0.58)	4.1.2.1 (1)	Susceptible (1)	>64.0	16.0	16.0	8.0	8.0	4.0	16.0	4.0	16.0	2.0	32.0	8.0	>64.0	8.0	-2.880
			965343	G>C	L65V	1 (0.58)	4.1.1.3 (1)	MDR (1)	16.0	0.3	2.0	0.5	8.0	8.0	4.0	0.5	4.0	1.0	1.0	0.3	64.0	4.0	-0.250
	Rv1009	*rpfB*	1128637	C>T	P183S	1 (0.58)	4.8 (1)	Poly-resistant (1)	>64.0	4.0	16.0	4.0	8.0	4.0	8.0	1.0	8.0	1.0	8.0	1.0	64.0	8.0	-5.719
			1128883	G>A	V265M	2 (1.16)	2.2.1.1 (2)	Susceptible (1)<br>Pre-XDR (1)	>64.0	22.6	22.6	5.7	8.0	4.0	22.6	2.8	32.0	2.8	32.0	2.8	>64.0	11.3	-2.122
	Rv1477	*ripA*	1667897	A>G	N303S	1 (0.58)	4.3.4.2 (1)	Mono-resistant (1)	16.0	0.3	2.0	1.0	8.0	2.0	4.0	0.5	2.0	1.0	0.5	0.3	32.0	4.0	-1.340
			1668082	G>C	A365P	2 (1.16)	2.2.1 (2)	MDR (2)	>64.0	2.8	16.0	4.0	8.0	4.0	8.0	1.0	16.0	1.0	8.0	0.7	>64.0	16.0	-3.081
			1668375	G>A	M462I	1 (0.58)	4.3.4.2 (1)	MDR (1)	64.0	1.0	8.0	2.0	4.0	2.0	2.0	0.5	4.0	0.5	4.0	0.5	32.0	4.0	-3.081
	Rv1478	*ripB*	1668582	T>G	V55G	1 (0.58)	4.3.4.1 (1)	Susceptible (1)	64.0	4.0	8.0	2.0	4.0	4.0	2.0	0.5	2.0	1.0	2.0	1.0	32.0	2.0	-5.565
	Rv1566c	-	1774149	C>T	G158S	1 (0.58)	4.8 (1)	Mono-resistant (1)	64.0	1.0	8.0	2.0	4.0	2.0	4.0	0.5	4.0	0.5	2.0	0.5	32.0	4.0	-4.005
	Rv1728c	-	1954333	A>C	L101W	2 (1.16)	4.8 (2)	Susceptible (2)	>64.0	5.7	32.0	16.0	11.3	8.0	8.0	1.4	8.0	1.0	11.3	5.7	32.0	5.7	-3.859
	Rv1884c	*rpfC*	2134215	T>C	H16R	86 (50.00)	4.3.3 (6)<br>4.3.4.1 (30)<br>4.3.4.2 (50)	Susceptible (42)<br>Mono or Poly-resistant (9)<br>MDR (22)<br>Pre-XDR (13)	31.5	1.1	8.5	2.3	5.5	3.2	2.5	0.5	3.3	0.6	2.5	0.6	23.6	3.1	-0.091
			2134215	TGTCT>CGTCA	RH15*	1 (0.58)	4.3.4.2 (1)	MDR (1)	8.0	0.3	2.0	0.5	4.0	2.0	2.0	0.5	4.0	0.5	1.0	0.3	32.0	4.0	-334.905
	Rv2190c	-	2452452	G>A	T274M	1 (0.58)	3 (1)	MDR (1)	>64.0	4.0	16.0	2.0	8.0	4.0	16.0	2.0	32.0	2.0	16.0	1.0	>64.0	16.0	-0.863
			2452756	C>A	A173S	16 (9.30)	4.3.4.2 (16)	Susceptible (3)<br>Mono or Poly-resistant (1)<br>MDR (5)<br>Pre-XDR (7)	41.5	1.0	7.7	2.4	5.0	3.5	2.5	0.4	3.5	0.6	3.1	0.6	21.7	2.8	-2.883
			2452983	G>A	A97V	1 (0.58)	4.1.2 (1)	Susceptible (1)	64.0	4.0	8.0	4.0	8.0	4.0	8.0	1.0	8.0	1.0	8.0	2.0	64.0	4.0	-2.411
			2453025	G>A	A83V	7 (4.07)	4.1.1.3 (7)	Susceptible (4)<br>MDR (3)	47.6	0.9	5.4	1.1	4.9	3.6	3.0	0.6	5.4	0.8	2.4	0.6	47.6	4.4	-1.920
			2453256	C>A	R6M	2 (1.16)	4.1.1.3 (2)	Susceptible (2)	45.3	1.0	2.8	1.0	4.0	4.0	2.8	0.7	8.0	0.7	2.8	0.7	64.0	5.7	-0.539
	Rv2450c	*rpfE*	2751804	C>T	R126Q	172 (100.00)	All	All	48.3	1.8	10.7	3.2	5.5	3.3	3.9	0.7	5.3	0.8	4.0	0.8	33.3	4.2	3.695
	Rv3915	*cwlM*	4403244	G>C	S18T	1 (0.58)	4.1.2.1 (1)	MDR (1)	32.0	1.0	4.0	4.0	8.0	4.0	16.0	1.0	16.0	2.0	8.0	1.0	64.0	8.0	-2.205
			4403495	C>G	P102A	1 (0.58)	4.3.4.1 (1)	Susceptible (1)	32.0	1.0	8.0	4.0	4.0	2.0	2.0	0.5	2.0	0.5	2.0	0.5	16.0	2.0	-4.373
			4403900	A>G	M237V	50 (29.07)	4.3.4.2 (50)	Susceptible (14)<br>Mono or Poly-resistant (6)<br>MDR (17)<br>Pre-XDR (13)	25.6	0.9	6.2	1.7	5.2	3.1	2.6	0.5	3.5	0.6	2.5	0.5	18.9	2.8	-3.597
			4403961	C>T	T257I	1 (0.58)	4.1.2.1 (1)	Susceptible (1)	32.0	1.0	4.0	2.0	4.0	4.0	2.0	0.5	4.0	0.5	4.0	0.3	16.0	2.0	1.658
Function	Locus Tag	Gene name	Genomic locus	Mutation	Effect in product	Number of isolates (%)	Sublineage	Drug-Resistance	AMX	AMX/CLA	CTX	CTX/CLA	FAR	FAR/CLA	BIA	BIA/CLA	DOR	DOR/CLA	MEM	MEM/CLA	ETP	ETP/CLA	Provean Score[a]
									48.3[b]	1.8[b]	10.7[b]	3.2[b]	5.5[b]	3.3[b]	3.9[b]	0.7[b]	5.3[b]	0.8[b]	4.0[b]	0.8[b]	33.3[b]	4.2[b]	
Cell division																							
	Rv0008c	*cwsA*	11879	A>G	S145P	172 (100.00)	All	All	48.3	1.8	10.7	3.2	5.5	3.3	3.9	0.7	5.3	0.8	4.0	0.8	33.3	4.2	6.533
			11929	G>C	S128W	1 (0.58)	4.8 (1)	Poly-resistant (1)	>64.0	4.0	16.0	4.0	8.0	4.0	8.0	1.0	8.0	1.0	8.0	1.0	64.0	8.0	-3.824
			12015	G>C	S99R	2 (1.16)	4.7 (2)	Susceptible (2)	>64.0	2.8	22.6	8.0	2.8	2.8	4.0	1.0	5.7	1.0	4.0	1.0	45.3	8.0	4.330
	Rv2719c	*chiZ*	3031090	G>A	Q150*	2 (1.16)	2.2.1 (2)	MDR (2)	>64.0	2.8	16.0	4.0	8.0	4.0	8.0	1.0	16.0	1.0	8.0	0.7	>64.0	16.0	-38.715
			3031168	A>G	Y124H	12 (6.98)	2.2.1 (8)<br>2.2.1.1 (2)<br>3 (1)<br>6 (1)	Susceptible (6)<br>MDR (5)<br>Pre-XDR (1)	>64.0	9.5	26.9	8.0	7.6	2.8	15.1	1.3	22.6	1.6	18.0	1.4	>64.0	10.7	0.863
	Rv2748c	*ftsK*	3061615	T>C	M298V	6 (3.49)	4.3.3 (6)	Susceptible (5)<br>MDR (1)	50.8	1.8	25.4	6.3	6.3	3.6	3.2	0.7	4.0	0.6	3.2	0.6	28.5	4.0	-0.703
			3062139	A>G	M123T	4 (2.33)	4.4.1.1 (4)	Susceptible (4)	>64.0	5.7	26.9	11.3	6.7	4.8	22.6	1.7	22.6	1.7	16.0	2.0	>64.0	9.5	-1.710
			3062386	C>T	G41S	1 (0.58)	4.3.4.1 (1)	Mono-resistant (1)	16.0	0.5	4.0	1.0	4.0	2.0	1.0	0.5	1.0	0.3	1.0	0.3	16.0	4.0	-0.527
	Rv3102c	*ftsE*	3471323	C>G	R16P	1 (0.58)	6 (1)	Susceptible (1)	>64.0	2.0	16.0	4.0	32.0	2.0	8.0	1.0	64.0	2.0	8.0	0.5	4.0	1.0	-6.658
	Rv3610c	*ftsH*	4051823	T>C	D354G	4 (2.33)	4.4.1.1 (4)	Susceptible (4)	>64.0	5.7	26.9	11.3	6.7	4.8	22.6	1.7	22.6	1.7	16.0	2.0	>64.0	9.5	-5.429
			4052598	T>A	S96C	2 (1.16)	4.7 (2)	Susceptible (2)	45.3	1.4	5.7	1.4	4.0	2.0	2.8	0.3	5.7	0.4	2.8	0.4	32.0	4.0	-2.703
	Rv3660c	*ssd*	4098382	C>A	R256L	1 (0.58)	4.8 (1)	Susceptible (1)	64.0	1.0	4.0	2.0	8.0	4.0	8.0	1.0	8.0	1.0	4.0	2.0	64.0	4.0	-5.115
			4098514	T>C	D212G	1 (0.58)	6 (1)	Susceptible (1)	>64.0	2.0	16.0	4.0	32.0	2.0	8.0	1.0	64.0	2.0	8.0	0.5	4.0	1.0	-0.576
			4098556	G>A	S198L	1 (0.58)	4.8 (1)	Susceptible (1)	32.0	0.3	4.0	2.0	8.0	4.0	4.0	1.0	4.0	1.0	1.0	0.3	32.0	4.0	-4.497

Table S3. Core-SNVs in beta-lactamase, transpeptidase or cell wall biosynthesis genes and geometric mean MIC for the strains with each considered mutation. Only non-synonymous mutations are shown. The number of strains in each sublineage and drug-resistance profile for the considered SNVs is represented between parentheses. The asterisk symbol (*) signifies a translation termination (stop) codon.
[a]PROVEAN scores below the -2.5 cut-off were predicted to have a deleterious impact on protein function. [b]Global geometric mean MIC for all clinical strains. AMX, amoxicillin; BIA, biapenem; CLA, clavulanate; CTX, cefotaxime; DOR, doripenem; ETP, ertapenem; FAR, faropenem; MEM, meropenem.
